# Subjective status and perceived legitimacy across countries

**DOI:** 10.1002/ejsp.2694

**Published:** 2020-06-30

**Authors:** Mark J. Brandt, Toon Kuppens, Russell Spears, Luca Andrighetto, Frederique Autin, Peter Babincak, Constantina Badea, Jaechang Bae, Anatolia Batruch, Julia C. Becker, Konrad Bocian, Bojana Bodroža, David Bourguignon, Marcin Bukowski, Fabrizio Butera, Sarah E. Butler, Xenia Chryssochoou, Paul Conway, Jarret T. Crawford, Jean‐Claude Croizet, Soledad de Lemus, Juliane Degner, Piotr Dragon, Federica Durante, Matthew J. Easterbrook, Iniobong Essien, Joseph P. Forgas, Roberto González, Sylvie Graf, Peter Halama, Gyuseog Han, Ryan Y Hong, Petr Houdek, Eric R. Igou, Yoel Inbar, Jolanda Jetten, William Jimenez Leal, Gloria Jiménez‐Moya, Jaya Kumar Karunagharan, Anna Kende, Maria Korzh, Simon M. Laham, Joris Lammers, Li Lim, Antony S. R. Manstead, Janko Međedović, Zachary J. Melton, Matt Motyl, Spyridoula Ntani, Chuma Kevin Owuamalam, Müjde Peker, Michael J. Platow, J. P. Prims, Christine Reyna, Mark Rubin, Rim Saab, Sindhuja Sankaran, Lee Shepherd, Chris G. Sibley, Agata Sobkow, Bram Spruyt, Pernille Stroebaek, Nebi Sümer, Joseph Sweetman, Catia P. Teixeira, Claudia Toma, Adrienn Ujhelyi, Jojanneke van der Toorn, Alain van Hiel, Alejandro Vásquez‐Echeverría, Alexandra Vazquez, Michelangelo Vianello, Marek Vranka, Vincent Yzerbyt, Jennifer L. Zimmerman

**Affiliations:** ^1^ Tilburg University Tilburg The Netherlands; ^2^ University of Groningen; ^3^ University of Genova; ^4^ CeRCA Université de Poitiers CNRS; ^5^ University of Presov; ^6^ Université Paris Nanterre; ^7^ Gwangju Welfare Foundation; ^8^ University of Lausanne; ^9^ University of Osnabrueck; ^10^ Sopot Faculty of Psychology SWPS University of Social Sciences and Humanities; ^11^ Department of Psychology Faculty of Philosophy University of Novi Sad Novi Sad Serbia; ^12^ University of Lorraine; ^13^ Jagiellonian University; ^14^ College of DuPage; ^15^ Panteion University of Social and Political Sciences; ^16^ Florida State University; ^17^ The College of New Jersey; ^18^ Université Clermont‐Auvergne et CNRS; ^19^ University of Granada; ^20^ Hamburg University; ^21^ University of Milano‐Bicocca; ^22^ University of Sussex; ^23^ FernUniversität in Hagen; ^24^ University of New South Wales; ^25^ Pontificia Universidad Católica de Chile; ^26^ Czech Academy of Sciences; ^27^ Slovak Academy of Sciences; ^28^ Chonnam National University; ^29^ National University of Singapore; ^30^ University of Economics in Prague; ^31^ University of Limerick; ^32^ University of Toronto; ^33^ University of Queensland; ^34^ Universidad de los Andes; ^35^ The University of Nottingham Malaysia Campus; ^36^ ELTE Eötvös Loránd University Budapest Hungary; ^37^ Ural State Law University; ^38^ University of Melbourne; ^39^ University of Cologne; ^40^ Cardiff University; ^41^ Institute of Criminological and Sociological Research Belgrade Serbia; ^42^ University of Illinois Chicago IL USA; ^43^ Civil Politics; ^44^ MEF University; ^45^ The Australian National University; ^46^ DePaul University; ^47^ The University of Newcastle Australia; ^48^ American University of Beirut; ^49^ University of Warsaw; ^50^ Northumbria University; ^51^ University of Auckland; ^52^ Wroclaw Faculty of Psychology SWPS University of Social Sciences and Humanities; ^53^ Vrije Universiteit Brussel; ^54^ University of Copenhagen; ^55^ Sabanci University; ^56^ University of Exeter; ^57^ Universite libre de Bruxelles; ^58^ Utrecht University; ^59^ Leiden University; ^60^ University of Gent; ^61^ Universidad de la República Montevideo Uruguay; ^62^ Universidad Nacional de Educación a Distancia; ^63^ University of Padova; ^64^ Charles University; ^65^ Université catholique de Louvain

**Keywords:** legitimacy, social identity, status, system justification

## Abstract

The relationships between subjective status and perceived legitimacy are important for understanding the extent to which people with low status are complicit in their oppression. We use novel data from 66 samples and 30 countries (*N* = 12,788) and find that people with higher status see the social system as more legitimate than those with lower status, but there is variation across people and countries. The association between subjective status and perceived legitimacy was never negative at any levels of eight moderator variables, although the positive association was sometimes reduced. Although not always consistent with hypotheses, group identification, self‐esteem, and beliefs in social mobility were all associated with perceived legitimacy among people who have low subjective status. These findings enrich our understanding of the relationship between social status and legitimacy.

## 1 INTRODUCTION

Scholars across the social sciences have debated whether or not people with low social status are complicit in their oppression. On the one hand, unequal social systems tend to be relatively stable across time, suggesting some degree of passivity, if not complicity among the populace; however, on the other hand, social movements aimed at disrupting or altering social systems are often spearheaded by the very people disaffected by existing status arrangements. The *status‐legitimacy hypothesis* (as coined by Brandt, 2013; Jost, Banaji, & Nosek, 2004; Jost & Hunyady, 2003; Jost, Pelham, Sheldon, & Ni Sullivan, 2003) predicts that people with lower status will, at least in some conditions, be more likely to see the social system as legitimate than people with higher status.^1^A related hypothesis predicts that lower power will be associated with higher levels of perceived legitimacy (van der Toorn et al., 2015). Our data also test this hypothesis; however, to sharpen the focus of the manuscript at the request of reviewers we have moved the power related analyses and discussion to supplemental materials. In this article, we examine the conditions under which this hypothesis finds support. We used a novel, 30‐country study on the association between subjective status and perceived legitimacy. We had two aims. First, we aimed to understand whether and when people with lower subjective status perceive higher levels of legitimacy than people with higher subjective status. Second, we aimed to understand what types of factors are associated with perceived legitimacy for people with low subjective status.

### 1.1 Status‐legitimacy hypothesis

The logic behind the status‐legitimacy hypothesis is that people have motivations to see themselves, their groups, and their larger social systems in a positive light (for full details see Jost et al., 2003). For people with high social status, all of these motivations are generally consistent with one another; however, for people with low social status who are disadvantaged by the social system, seeing the self and one's group as positive conflicts with the motivation to see the social system as positive. To resolve this psychological conflict people with low social status may, under some conditions, legitimize the social system *more* than people with high social status.

Scholars have debated the status‐legitimacy hypothesis, testing the hypothesis several times with mixed results (find support: e.g., Henry & Saul, 2006; Li, Yang, Wu, & Kou, 2020; Sengupta, Osborne, & Sibley, 2015; see Jost, 2017 for a recent summary of relevant work; do not find support: e.g., Brandt, 2013; Caricati, 2017; Caricati & Lorenzi‐Cioldi, 2012; Vargas‐Salfate, Paez, Liu, Pratto, & Gil de Zúñiga, 2018; Zimmerman & Reyna, 2013). This work suggests that there is still broad scholarly interest in how status and legitimacy are related. We aim to add two things to this literature. With *Approach 1*, we test potential individual‐ and societal‐level *moderators* of the associations between *different levels* of subjective status and perceived legitimacy (i.e., a relative focus). Under what conditions and among people with what kinds of perceptions and psychological characteristics is there evidence for the status‐legitimacy hypothesis (cf. McGuire, 2013)? With *Approach 2*, we focus on people with *low* levels of social status and tested potential predictors of perceived legitimacy among this group. Unlike the first approach, Approach 2 does not focus on whether the predictors differ from people with high levels of status.

### 1.2 Approach 1: Testing moderators of the association between subjective status and perceived legitimacy

The status‐legitimacy hypothesis follows from the idea that people are addressing the threatening feelings of aversive anxiety and arousal resulting from psychological conflict. People alter their behaviors, beliefs, and perceptions in order to address this threat, and when the feelings of threat are assuaged they are less likely to alter their behaviors, beliefs, and perceptions (for reviews see Jonas et al., 2014; Proulx, Inzlicht, & Harmon‐Jones, 2012). Factors that make social hierarchy appear less consequential in material or psychological terms (we discuss several factors below) should reduce the effects of low subjective status on the anxious arousal that results from status‐based dissonance. Therefore, this reasoning suggests that when people's feelings of threat are addressed, the status‐legitimacy link will be positive (i.e., low status people will be less inclined to legitimize the system as a way to assuage feelings of threat). Conversely, when people's anxious arousal is exacerbated, the status‐legitimacy link will be negative (i.e., low status people will see the system as more legitimate).

#### 1.2.1 Moderators that reduce threat

There are potentially many factors that can mitigate anxious arousal. We focus on factors that have appeared in the system justification and social identity literatures because these literatures have been the focus of debates on this issue (for a recent example see Jost, 2019; Owuamalam, Rubin, & Spears, 2019). The most obvious factor is the identification with and salience of a valued group, which is one way social identity theorists suggest people address anxious arousal (Hogg, 2014) or cope with the threat of having a low status (McMahon & Watts, 2002; Tajfel & Turner, 1979). Consistent with this, the original paper proposing the status‐legitimacy hypothesis argued that if group interests are salient and accessible (something that is likely to be correlated with identification), then support for the status‐legitimacy hypothesis is unlikely to emerge (Jost et al., 2003, 2004). Similarly, people with high self‐esteem and positive self‐views make plans, have high levels of personal agency, and high levels of self‐certainty (e.g., Campbell, 1990; Harter, 1978), suggesting that they are less likely to be psychologically affected by uncontrollability and similar types of threats associated with low status (see Laurin, Kay, & Landau, 2018 or Schoel, Bluemke, Mueller, & Stahlberg, 2011 for a similar argument in different domains). This is consistent with the idea that self‐esteem should push against system justification motivations for low status people (cf. Jost, Gaucher, & Stern, 2015, p. 330).^2^Social identity theory and system justification theory have also both used self‐esteem as an outcome variable. Although this is interesting, it is not the focus of our investigation.


#### 1.2.2 Structural moderators that increase threat through dissonance

Other factors can exacerbate feelings of anxious arousal by increasing the amount of dissonance people experience. The most direct prediction comes from assessing the contradictory cognitions that could increase dissonance. The dissonance that people experience from being in a low status position might emerge “from the contradictory cognitions that (a) the system is putting me (and my group) at a disadvantage, and (b) through our acquiescence, my group and I are contributing to the stability of the system” (Jost et al., 2003, p. 16). Therefore, people who recognize that they are not doing enough to mitigate inequality should experience more dissonance. This recognition should increase perceived legitimacy for people who are low status and is part of the contradictory cognitions originally predicted to cause lower status people to see the system as more legitimate than higher status people do.

The amount of civil liberties, meritocratic culture, and inequality within a country could also affect feelings of dissonance. Countries and contexts with more civil liberties and more meritocratic cultures increase the amount of dissonance people low in status experience by implying they have choice and control over their outcomes (Jost et al., 2003, p. 17). Inequality may increase the conflict between self/group motivations and acceptance of the system for low status group members (e.g., Henry & Saul, p. 376). It may also create the impression that people have less control over their place within society. Both factors should increase the amount of anxious uncertainty and increase the necessity of rationalizing the system. No support was found for these predictions in some prior studies (Brandt, 2013; Caricati, 2017; Caricati & Lorenzi‐Cioldi, 2012; Trump & White, 2018; for one exception on one measure see Vargas‐Salfate et al., 2018).

#### 1.2.3 Structural moderators that affect threat

Structural factors, including levels of inequality or the stability of the hierarchy, influence what types of options people perceive that they have and their place within the society. These perceptions may affect people's perceptions of the whole system. For example, to the extent that inequality exacerbates feelings of hierarchy and the threatening feelings of low status, as well as making status differences more salient, this should further motivate challenges to the inequality by the low status group (Tajfel & Turner, 1979). This is the opposite of the inequality prediction above. When looking at the stability of the hierarchy, one of the traditional predictions of social identity theory is that low status group members are less likely to seek social change when the status hierarchy is perceived as stable, compared to when the hierarchy is perceived as unstable (Ellemers, van Knippenberg, & Wilke, 1990; Tajfel & Turner, 1979). That is, when the status hierarchy is stable, there is little scope and hope for social change (i.e., no cognitive alternative to the status quo), and people are more likely to accept the system as legitimate. This hypothesis is also consistent with work finding that system stability increases a system justification motivation (Laurin, Gaucher, & Kay, 2013).

#### 1.2.4 Summary

We have identified several potential moderators that might help us predict when we are more or less likely to find support for the status‐legitimacy hypothesis. These moderators are expressed as individual hypotheses in Table 1. In the cross‐national study that follows, we test these eight hypotheses.

**TABLE 1:** Summary of the moderator hypotheses tested in this investigation (Approach 1)

Variable	Moderator hypothesis
*Reduce threat*	
Group Identification	*Identification‐Moderation Hypothesis*: The link between status and legitimacy will be negative for people low in group identification and positive for people high in group identification.
Self‐Esteem	*Self‐Esteem‐Moderation Hypothesis*: The link between status and legitimacy will be negative for people low in self‐esteem and positive for people high in self‐esteem.
*Increase threat through dissonance*	
Inequality contribution	*Inequality Contribution‐Moderation Hypothesis*: The link between status and legitimacy will be negative for people who see themselves as contributing to inequality and positive for people who do not see themselves as contributing to inequality.
Civil Liberties	*Civil Liberties Hypothesis*: The link between status and legitimacy will be negative in countries with high levels of civil liberties and positive for people in countries with low levels of civil liberties.
Meritocracy	*Meritocracy Hypothesis*: The link between status and legitimacy will be negative in countries with meritocratic cultures and positive for people in countries with less meritocratic cultures.
Inequality	*System Justification Theory (SJT) Inequality Hypothesis*: The link between status and legitimacy will be negative in countries with high levels of inequality and positive for people in countries with low levels of inequality.
*Structural Factors That Affect Threat*	
Inequality	*Social Identity Theory (SIT) Inequality Hypothesis*: The link between status and legitimacy will be negative in countries with low levels of inequality and positive for people in countries with high levels of inequality.
Stability	*Stability‐Moderator Hypothesis*: The link between status and legitimacy will be negative when people see the status hierarchy as stable and positive when people see the status hierarchy as unstable.

### 1.3 Approach 2: Finding the predictors of perceived legitimacy for people with *lower* levels of subjective status

In addition to searching for moderators, we also ask what predicts perceived legitimacy for people with low levels of social status. Put another way, assuming that people with low levels of subjective status vary in the extent to which they see the system as legitimate, what predicts this variation? In contrast to the prior section, this line of questioning does not necessarily imply moderation effects, as the predictors of perceived legitimacy for lower status groups *could* be the same as the predictors of perceived legitimacy for higher status groups (moderation is possible, but not necessary). This approach moves away from the explicit status comparisons of the status‐legitimacy hypothesis and is consistent with the broader questions that inspired this hypothesis: Do people see systems that oppress them as just and legitimate, and if so under what conditions? We focus on four different variables that might predict perceived legitimacy for people with lower levels of social status (see Table 2).

**TABLE 2:** Summary of the predictor hypotheses tested in this investigation (Approach 2)

Variable	Predictor hypothesis
Social mobility	*Social Mobility‐Legitimacy Hypothesis*: People with low status who see the system as having high social mobility will be more likely to see the system as legitimate.
Stability	*Stability‐Legitimacy Hypothesis*: People with low status who see the status hierarchy as stable will be more likely to see the system as legitimate.
Identification	*Identification‐Legitimacy Hypothesis*: People with low status who have high group identification will be less likely to see the system as legitimate.
Self‐esteem	*Self‐Esteem Legitimacy Hypothesis*: People with low status who have high self‐esteem will be less likely to see the system as legitimate.

Some work suggests that people desire to live in social systems in which there is upward mobility and the system is relatively stable (Laurin et al., 2013; Martorana, Galinsky, & Rao, 2005). Consistent with this, people who perceived no possibilities to move up to a higher status group are less likely to justify the system and more likely to engage in collective action (Day & Fiske, 2017; Ellemers et al., 1990; Mandisodza, Jost, & Unzueta, 2006; Tajfel, 1981; Wright, Taylor, & Moghaddam, 1990). Similarly, systems with more stable social hierarchies are seen as more legitimate (Laurin et al., 2013) and are less likely to trigger efforts to change the system (Bettencourt, Charlton, Dorr, & Hume, 2001; Ellemers et al., 1990; Tajfel & Turner, 1979). This suggests that when people see the system as providing social mobility and stability, they are more likely to see the system as legitimate.

When people's group‐ and self‐interests are prioritized, it may be less likely that people perceive the system as legitimate if they or their group are not benefitting from that system. That is, higher levels of group identification and self‐esteem may both be associated with lower levels of perceived system legitimacy among people with low social status. This follows from the idea that group‐ and self‐interest motivations are negatively related to system‐related motivations among low status groups (Jost et al., 2004). It is also consistent with the idea that the effects of group‐ and self‐interests—when sufficiently strong—may be more prominent than the effects of system‐interests (Jost et al., 2003, 2004, 2011) and with the finding that people with low levels of group identification may be more likely to accept the current situation (Rubin & Hewstone, 2004; Spears, Jetten, & Doosje, 2001). According to these ideas, people with lower subjective status who have high group identification or who have high self‐esteem will be less likely to see the system as legitimate.

From our reading of the literature, the predictions in the prior paragraph seem most consistent with a straightforward extension of social identity theory's work on group identification to research on perceived legitimacy. However, it is important to note that recent work by some scholars (and co‐authors of this article) has predicted the *opposite*, at least as a function of an additional qualifier. A series of papers by Owuamalam and colleagues have argued that *high* group identifiers and people under conditions of *high* group salience may be likely to see the system as legitimate *to the extent that it is seen as one that affords the group collective social mobility in the long term* and allows them to eventually achieve social change as a group (e.g., due to longer‐term status instability; Owuamalam, Rubin, & Spears, 2016, 2019; Owuamalam, Rubin, Spears, & Weerabangsa, 2017; for a countervailing view see Jost, 2019). In short, using the system rather than rejecting it, can be seen as a viable vehicle for group interests, especially for high identifiers. This idea can also be tested with our data and essentially predicts the opposite of the predictions in the prior paragraph.

#### 1.3.1 Summary

We identified several potential predictors of perceived legitimacy for people with lower social status that might help us understand the reasons some people with low social status perceive the social system as legitimate. Some predictors indicate that the system is fulfilling the person's goals and other predictors indicate that personal and group goals are prioritized. These hypotheses are specified individually in Table 2. In the cross‐national study that follows, we test these four hypotheses.

### 1.4 An international, multi‐lab approach

We conducted a cross‐country study on the association between subjective status and perceived legitimacy that allowed us to test for moderators of the status‐legitimacy relationships, as well as to understand what predicts perceived legitimacy for people with lower levels of subjective status.^3^Recently, system justification theorists have proposed that a low sense of power, rather than status, is associated with greater perceived legitimacy. Whereas status indicates the amount of prestige and respect a person or group is accorded in the system, power indicates the amount of control a person has over valued resources (Magee & Galinsky, 2008). Trusting and legitimizing outside sources of control (e.g., governments) can help restore people's sense of control, something that is lacking with low feelings of power (van der Toorn et al., 2015; cf. Friesen et al., 2014; Kay et al., 2008). By perceiving the system as legitimate, people with low feelings of power can regain some feelings of control. We therefore simultaneously tested all hypotheses for interpersonal sense of power. Complete results are in the supplemental materials. We consider three types of effects:

To assess moderators (Approach 1):

1. We test the interactions between status and the proposed moderator variables. If the interaction is significant, we test whether the effect of subjective status is negative (consistent with the status‐legitimacy hypothesis) at the predicted levels of the moderator variables (e.g., when identification is low).

The assessment of predictors of perceived legitimacy among people with *low* status will take two steps (Approach 2):

2a. We examine the main effects of the predictors on perceived legitimacy. Because the hypotheses about predictors of perceived legitimacy are not specific to people with lower levels of subjective status (e.g., social mobility could predict perceived legitimacy for people with both high and low status), this main effect analysis tells us if there is an average effect across the sample.

2b. When there is a significant interaction between the predictor and status, we examine whether the predictor still has the predicted significant effect (e.g., a positive effect of social mobility) for people with lower levels of status. This will tell us whether the effect of the predictor is specific to people with low subjective status.

## 2 METHOD

### 2.1 Participants and procedure

Sixty‐six distinct samples were collected by researchers from 30 countries. We aimed for 150 participants per sample, so that we had at least 150 participants per country to give us approximately 80% power to detect a small to medium effect (i.e., *r* = .22) within each sample. Data collection was not continued after analysis. To ensure that respondents were part of the social and political system, we only included participants who indicated they were either born in the country or had lived in the country for six or more years and so not every sample resulted in 150 participants.^4^Six or more years was chosen to ensure that participants who were international or exchange students were not included in the sample. All exclusions are reported. Countries, samples, type of sample, proportion of men, mean age, and sample size are presented in Table 3. Samples included a mix of student samples, community samples, and samples with both students and members of the community (the latter two were both considered non‐student samples for the sake of analyses). Samples are primarily samples of university students, although some include community samples from Mechanical Turk (USA2), community email lists (e.g., USA6), or a representative sample (NLD3). Multiple samples per country helps guard against the possibility that results for any particular country are dependent on one particular sample. Research was conducted in accordance with APA and national guidelines. Data, code, and materials are available at the following link: https://osf.io/5uxc7/.

**TABLE 3:** Sample demographic information. Sorted in alphabetical order by country

Sample	Country	Type	Proportion Male	*M* age	*N*
AUS1	Australia	Student	0.27	21.4	209
AUS2	Australia	Student	0.34	19.5	114
AUS3	Australia	Student	0.34	19.2	80
AUS4	Australia	Student	0.29	20.1	163
AUS5	Australia	Student	0.32	23.2	222
BEL1	Belgium	Student	0.35	20.0	623
BEL2	Belgium	Student	0.54	21.7	137
BEL3	Belgium	Student	0.28	19.3	194
BEL4	Belgium	Student	0.24	21.9	91
CAN1	Canada	Student	0.33	18.7	180
CHL1	Chile	Student	0.28	20.9	156
COL1	Colombia	Student	0.27	19.9	139
CZE1	Czech Republic	Non‐Student	0.23	27.5	154
CZE2	Czech Republic	Non‐Student	0.29	23.7	241
CZE3	Czech Republic	Non‐Student	0.30	24.8	311
DNK1	Denmark	Student	0.16	24.0	162
FRA1	France	Student	0.31	21.2	303
FRA2	France	Student	0.11	20.8	163
FRA3	France	Student	0.46	18.6	180
DEU1	Germany	Student	0.36	25.3	50
DEU2	Germany	Student	0.18	22.6	133
DEU3	Germany	Student	0.22	22.7	151
DEU4	Germany	Non‐Student	0.81	37.2	89
GBR1	Great Britain	Student	0.14	19.8	213
GBR2	Great Britain	Student	0.07	19.1	138
GBR3	Great Britain	Student	0.18	19.6	169
GBR4	Great Britain	Student	0.08	19.1	118
GRC1	Greece	Non‐Student	0.49	35.9	444
HUN1	Hungary	Student	0.22	20.2	144
IND1	India	Non‐Student	0.74	31.2	449
IRL1	Ireland	Student	0.46	24.6	145
ITA1	Italy	Student	0.47	43.0	103
ITA2	Italy	Student	0.47	44.0	103
ITA3	Italy	Student	0.05	26.9	109
LBN1	Lebanon	Student	0.51	18.9	204
MYS1	Malaysia	Student	0.43	20.8	146
MYS2	Malaysia	Non‐Student	0.44	24.6	63
NLD1	Netherlands	Student	0.26	19.8	184
NLD2	Netherlands	Student	0.19	20.0	232
NLD3	Netherlands	Non‐Student	0.49	40.4	766
NLD4	Netherlands	Student	0.20	21.2	176
NZL1	New Zealand	Student	0.18	21.0	180
POL1	Poland	Student	0.16	28.0	214
POL2	Poland	Non‐Student	0.20	23.2	160
POL3	Poland	Non‐Student	0.65	26.4	166
RUS1	Russia	Student	0.34	19.4	117
SRB1	Serbia	Student	0.24	20.3	159
SRB2	Serbia	Non‐Student	0.39	30.7	173
SGP1	Singapore	Student	0.27	19.9	196
SVK1	Slovakia	Student	0.19	22.6	268
SVK2	Slovakia	Non‐Student	0.49	46.7	166
KOR1	South Korea	Student	0.30	20.6	119
ESP1	Spain	Student	0.24	23.1	148
ESP2	Spain	Student	0.30	32.7	252
CHE1	Switzerland	Student	0.51	20.3	131
TUR1	Turkey	Student	0.30	20.5	122
TUR2	Turkey	Student	0.23	20.0	99
USA1	United States	Student	0.20	20.6	224
USA2	United States	Non‐Student	0.57	35.1	214
USA3	United States	Student	0.42	19.8	316
USA4	United States	Student	0.27	19.9	195
USA5	United States	Student	0.17	19.4	181
USA6	United States	Non‐Student	0.23	36.1	368
USA7	United States	Non‐Student	0.48	41.5	116
URY1	Uruguay	Student	0.28	22.2	169
URY2	Uruguay	Student	0.21	20.6	184

Participating labs used materials designed by the first three authors. These labs translated the materials and adjusted them when necessary for their language and cultural context (e.g., replacing “United States” in the system justification measure). Participants first completed measures of demographics, including the measure of subjective social status. They then completed measures about either their perceptions of themselves and their group's position in society (the moderators and predictors) or their perceptions of system legitimacy (the outcomes). The supplemental materials include a list of all measures and manipulations. Analyses include participants who are over 18 and have completed at least the subjective status and the system justification, trust in government, confidence in societal institutions, and legitimacy of the status hierarchy measures. The final sample included 12,788 participants (4,252 men, 8,478 women, 58 with missing responses, *M*_age_ = 25.3, *SD*_age_ = 10.7).

### 2.2 Key predictor variable: subjective status

To measure subjective social status, we used the MacArthur Scale of Subjective Social Status (Adler, Epel, Castellazzo, & Ickovics, 2000), modified to capture people's sense of status within their country. We chose this measure to allow easier comparisons across countries. Participants were asked to rate themselves on a ladder that ranged from 1 to 10, where 10 was high status and 1 was low status. The instructions for the measure read as follows:

Think of this ladder as representing where people stand in [country]. At the top of the ladder are people who are the best off—those who have the most money, the most education, and the most respected jobs. At the bottom are the people who are the worst off—who have the least money, least education, and the least respected jobs or no job. The higher up you are on this ladder, the closer you are to the people at the very top; the lower you are, the closer you are to the people at the very bottom. Please choose the number of the rung of the ladder where you think you stand at this time in your life, relatively to other people in [country].

This one‐item measure had adequate test‐retest reliability in prior samples (e.g., Operario, Adler, & Williams, 2004) and is correlated with objective measures of status (e.g., income; Goodman et al., 2003; Sakurai, Kawakami, Yamaoka, Ishikawa, & Hashimoto, 2010). Responses in our sample were above the midpoint (*M* = 6.03, *SD* = 1.53), but spanned the entire range of the measure. See supplemental materials for means across countries on this and all other individual‐level variables.

### 2.3 Key outcome variable: perceived legitimacy

We included four measures of perceived legitimacy and system justification. Multiple research groups studying the status‐legitimacy hypothesis have used all these measures (e.g., Brandt, 2013; Henry & Saul, 2006; Jost et al., 2003; Li et al., 2020). The 8‐item general system justification scale (Kay & Jost, 2003) includes items like “In general, I find society to be fair” and “Society is set up so that people usually get what they deserve” (*M* *α* = .78, *SD* *α* = .06, *α* range [.66, .85]; Scale *M* = −0.64; Scale *SD* = 1.14; −3 = *Disagree strongly* to +3 = *Agree strongly*).^5^For each scale, alpha or the correlation coefficient (for 2‐item scales) was calculated in each country separately. Mean, standard deviation, and range of alphas and correlation coefficients across countries are reported in the text.


The 4‐item trust in government scale often included in the American National Election Studies (2015; cf. Brandt, 2013) includes items like “How much of the time do you think you can trust the government to do what is right?” (1 = *None of the time*, 2 = *Some of the time*, 3 = *Most of the time*, 4 = *Just about always*) and “Would you say the government is pretty much run by a few big interests looking out for themselves or that it is run for the benefit of all the people?” (1 = *Few big interests*, 2 = *Benefit of all*). Items were first standardized using *z*‐scores and then averaged to form a scale (*M* *α* = .61, *SD* *α* = .17, *α* range [−.10, .78,]; Scale *M* = −0.02; Scale *SD* = 0.75).^6^The negative *α* (−.06) for the trust in government scale is found in Russia. The next lowest *α* is .20 in Lebanon.


The 7‐item measure assessing confidence in societal institutions adopted from the General Social Survey (2017), and used in Brandt (2013), was used to tap into perceptions of both governmental and economic systems. For each of seven institutions, participants are asked how much confidence they have in them (0 = *None at all*, 3 = *A great deal*). Institutions include the armed forces, the police, the courts, the governments of the country, congress, major companies, and banks and financial institutions (*M* *α* = .77, *SD* *α* = .04, α range [.63, .83,]; Scale *M* = 1.35; Scale *SD* = 0.55).

Finally, a 2‐item measure of the perceived legitimacy of the status hierarchy was used to capture perceived legitimacy in this specific domain (based on Mummendey, Kessler, Klink, & Mielke, 1999). The items were prefaced with “Differences in power and status between groups in [country] are...” and then participants rated the stems “…illegitimate” and “…unfair” on a scale ranging from −3 = *Disagree strongly* to 3 = *Agree strongly* (*M r* = .52, *SD r* = .14, *r* range [.33, .80]; Scale *M* = −0.83; Scale *SD* = 1.30). These items were measured in the same block of questions as the stability of the status hierarchy measure (see below).

### 2.4 Individual level measures of moderators and predictors

#### 2.4.1 Group identification

We measured group identification with a three‐item scale about participants’ identification with their social class. The scale was prefaced with, “The following questions are about people with a similar background and social class as yourself. Social class refers to people with similar opportunities in terms of income, education, and social standing, as in the ladder measure that you completed earlier.” The items were “I identify with people from my social class”, “I feel solidarity with my social class”, and “My social class is an important part of how I see myself”. Participants responded to these items on a scale ranging from −3 = *Disagree strongly* to 3 = *Agree strongly* (*M* *α* = .67, *SD* *α* = .10, *α* range [.38, .78]; Scale *M* = 0.51; Scale *SD* = 1.17).

#### 2.4.2 Self‐esteem

We measured self‐esteem with the validated single‐item measure (Robins, Hendin, & Trzesniewski, 2001). This measure reads “I have high self‐esteem” with answers that ranged from 0 = *Not at all* to 6 = *Very true of me* (Item *M* = 3.50; Item *SD* = 1.57).

#### 2.4.3 Social mobility

We measured perceptions of social mobility with six items. They included, “In general, people can easily get ahead in society”, “In general, people can climb the social ladder and be successful”, “People with a similar background and social class to my own can easily get ahead in society”, “It is easy for people with a similar background and social class to my own to climb the social ladder and be successful”, “I am motivated to climb up the social ladder”, and “I am able to climb up the social ladder”. All items were measure on a scale ranging from −3 = *Disagree strongly* to 3 = *Agree strongly* and had good reliability (*M* *α* = .72, *SD* *α* = .06, *α* range [.52, .81]; Scale *M* = 0.82; Scale *SD* = 0.93).

#### 2.4.4 Contribute to inequality

We created five items to measure whether people felt that they contributed to inequality and the stability of the system. One item, “I could do more to change differences in power and status between groups in society”, did not correlate as expected with the other items and substantially reduced the reliability of the scale (*α* = .58), so we omitted it. The remaining four items read “I contribute to keeping society the way it is”, “I contribute to maintaining the current social hierarchy”, “I don't do anything to change the current differences in power and status in society”, and “I am not trying to change the current differences in power and status in society”. All items were measured on a scale ranging from −3 = *Disagree strongly* to 3 = *Agree strongly* and the four remaining items created a reliable scale (*M* *α* = .71, *SD* *α* = .09, *α* range [.46, .84]; Scale *M* = −0.14; Scale *SD* = 1.12).

#### 2.4.5 Stability of the status hierarchy

We used a 2‐item measure of the perceived stability of the status hierarchy. This was based on measures used in studies from a social identity perspective (Mummendey et al., 1999). The items were prefaced with “Differences in power and status between groups in [country] are...” and then participants rated the stems “…difficult to change” and “…will remain stable over time” on a scale ranging from −3 = *Disagree strongly* to 3 = *Agree strongly* (*M r* = .31, *SD r* = .14, *r* range [−.06, .76]; Scale *M* = 0.82; Scale *SD* = 1.14).^7^The negative correlation (−.06) between the stability items is found in Malaysia. The next lowest correlation is .18 in Canada.


### 2.5 Societal level measures of moderators

We included three measures to assess societal level conditions. To capture the amount of civil liberties, we used the 2015 Civil Liberties subscale of the Democracy index created by the Economist Intelligence Unit (2016). This index uses a combination of survey data and expert ratings to estimate how democratically free individual countries are. It has been used in prior work testing similar questions (Brandt, 2013).

To assess the extent to which the culture holds meritocratic values, we combined four items from international surveys. The first two are the importance of ambition and hard work for getting ahead in life in the ISSP (2009). The third and fourth come from the sixth wave of the World Values Survey (2016). The third asks participants to respond to an item ranging from 1 = “Competition is good. It stimulates people to work hard and develop new ideas” to 10 = “Competition is harmful. It brings out the worst in people”. The fourth asks participants to respond to an item ranging from 1 = “In the long run, hard work usually brings better life” to 10 = “Hard work doesn't generally bring success—it's more a matter of luck and connections”. All four items were standardized, the third and fourth items were reverse scored, and all four items were combined to form a scale (*α* = .82).

To measure the objective inequality, we used the Gini index from 2013 and obtained from the World Bank (World Bank, 2016). For some countries, the 2013 data were not available, and in these cases the most recent data were included instead. The Gini index assesses the amount of income inequality within a region, with higher scores indicating greater inequality. It is a common measure of inequality within a society (e.g., Oishi, Kesebir, & Diener, 2011).

### 2.6 Covariates

We included three covariates for analyses that focused on the individual level: self‐reported age, gender (0 = women, 1 = men), and type of sample (0 = student, 1 = non‐student) to adjust for potential background influences. For the models that include predictors at the societal level of analysis, we included two country‐level covariates. We included the GDP per capita for each of the countries to control for overall wealth (World Bank, 2016). To control for broader regional trends (Kuppens & Pollet, 2014), we also included contrast codes for each of the continents represented in our data.

### 2.7 A note on coding

We coded all of the variables to range from 0 to 1. Multilevel regression coefficients are then the proportion difference in the outcome variable as one goes from 0 (minimum) to 1 (maximum) on the predictor variable. For example, a coefficient of .05 is a 5% difference in the outcome between people scoring the lowest and the highest on the predictor variable.

## 3 RESULTS

### 3.1 Preliminary analyses: the effects of subjective status on perceived legitimacy

**FIGURE 1:**
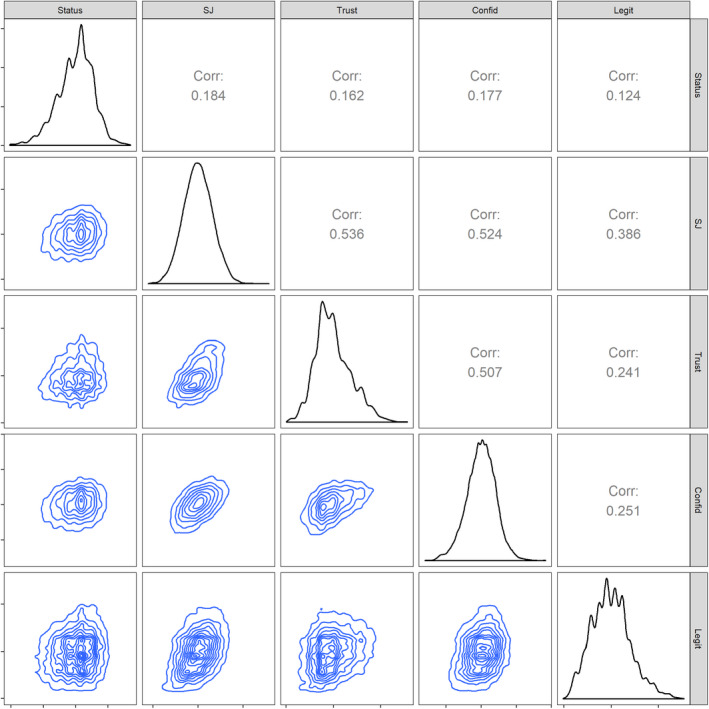
Correlations (above the diagonal) and density plots (below the diagonal) between subjective status, sense of power, and the outcome variables. All variables were country‐mean‐centered before calculating the correlations. Density plots for each individual variable are on the diagonal. Density plots are a variant of a histogram. The area below the diagonal uses density plots between two variables. SJ = System Justification, Trust = Trust in Government, Confid = Confidence in Societal Institutions, Legit = Legitimacy of the status hierarchy [Colour figure can be viewed at wileyonlinelibrary.com]

We use multilevel models to account for participants’ nesting within countries and within samples/labs. Models were estimated using lme4 (Bates, Mächler, Bolker, & Walker, 2015) and lmerTest (Kuznetsova, Brockhoff, & Christensen, 2016) packages in R (R Core Team, 2016). Because we are interested in the individual‐level association between subjective status and perceived legitimacy, we centered subjective status at the country‐mean (Enders & Tofighi, 2007). The correlations between the predictor variables and the measures of perceived legitimacy are in Figure 1 (created with GGally, Schloerke et al., 2016). The measures of system justification, trust, confidence, and legitimacy of the status hierarchy were moderately inter‐correlated (*M* *α* = .70, *SD* *α* = .06, *α* range [.56, .82]). To combine items, reduce the overall number of models, and facilitate generalization across measures, we nested these four measures within participants, resulting in a four‐level multilevel model: legitimacy measures nested in persons, nested in samples/labs, nested in countries. In short, this estimates the average effect across multiple measures of system legitimacy that have been used in the literature, while increasing measurement precision (see for similar suggestions, Gelman, 2018; McShane, Tackett, Böckenholt, & Gelman, 2019). It is analogous to conducting four studies and estimating the meta‐analytic estimate across the four studies.^8^We also conducted the analyses with only the system justification scale because this scale is perhaps the most well‐developed of the four scales and it was reliable in all of the countries. Conclusions in the main text are essentially unchanged. There are two exceptions. The negative interaction between stability and status was negative and non‐significant and the positive interaction between civil liberties and status was positive and non‐significant when only looking at the system justification scale. Importantly, the sizes of these coefficients were very similar to the coefficients in the models including all legitimacy measures, suggesting that the reduction in error variance when using additional data is the reason for the different conclusions when using all legitimacy measures or only the system justification scale.


First, we tested the main effect of subjective status on perceived legitimacy. This test conceptually replicates many prior tests of these hypotheses (e.g., Brandt, 2013; Henry & Saul, 2006; Jost et al., 2003). We regressed perceived legitimacy on country‐mean‐centered subjective status. We also included country‐mean‐centered sense of power in this analysis and all other analyses because it was part of our original analysis plan (see Appendix S1). People with higher subjective status are more likely to see the social system as legitimate compared to people with lower subjective status (*b* = .152, *SE* = 0.005, 95% CI [0.142, 0.162]). There is a 15% difference in perceived legitimacy between people with the highest and people with the lowest levels of subjective status. This is in contrast to the prediction of the status‐legitimacy hypothesis.

To test whether these results are impacted by covariates, we included age (country‐mean‐centered), gender (country‐mean‐centered), type of sample (grand mean‐centered), and the type of perceived legitimacy measure (contrast coded) as covariates, including the interactions between these contrast codes and status and sense of power.^9^We used the simr package in R (Green & MacLeod, 2016) to see how much power our model and sample size had to detect the effects of subjective status when *b* = .01, .02, .05, and .10. These analyses showed that we had adequate power to detect effects of at least .02 (i.e. a 2% difference in the outcome variable between the minimum and maximum of the predictor variable; power ≈ 49%, 97%, 100%, 100% respectively). This gives us the average effect of the primary predictors across the four measures of perceived legitimacy and controls for mean differences between the measures. The estimate for subjective status is nearly identical to the estimate without covariates (*b* = .151, *SE* = 0.005, 95% CI [0.141, 0.161]).^10^The variance for the intercept at each level of the multilevel models that included covariates was also calculated (Participants *σ* = .049, Labs *σ* = .006, Countries *σ* = .098, Residual *σ* = .18). Descriptively, there is more variation between countries than there is between labs.


**FIGURE 2:**
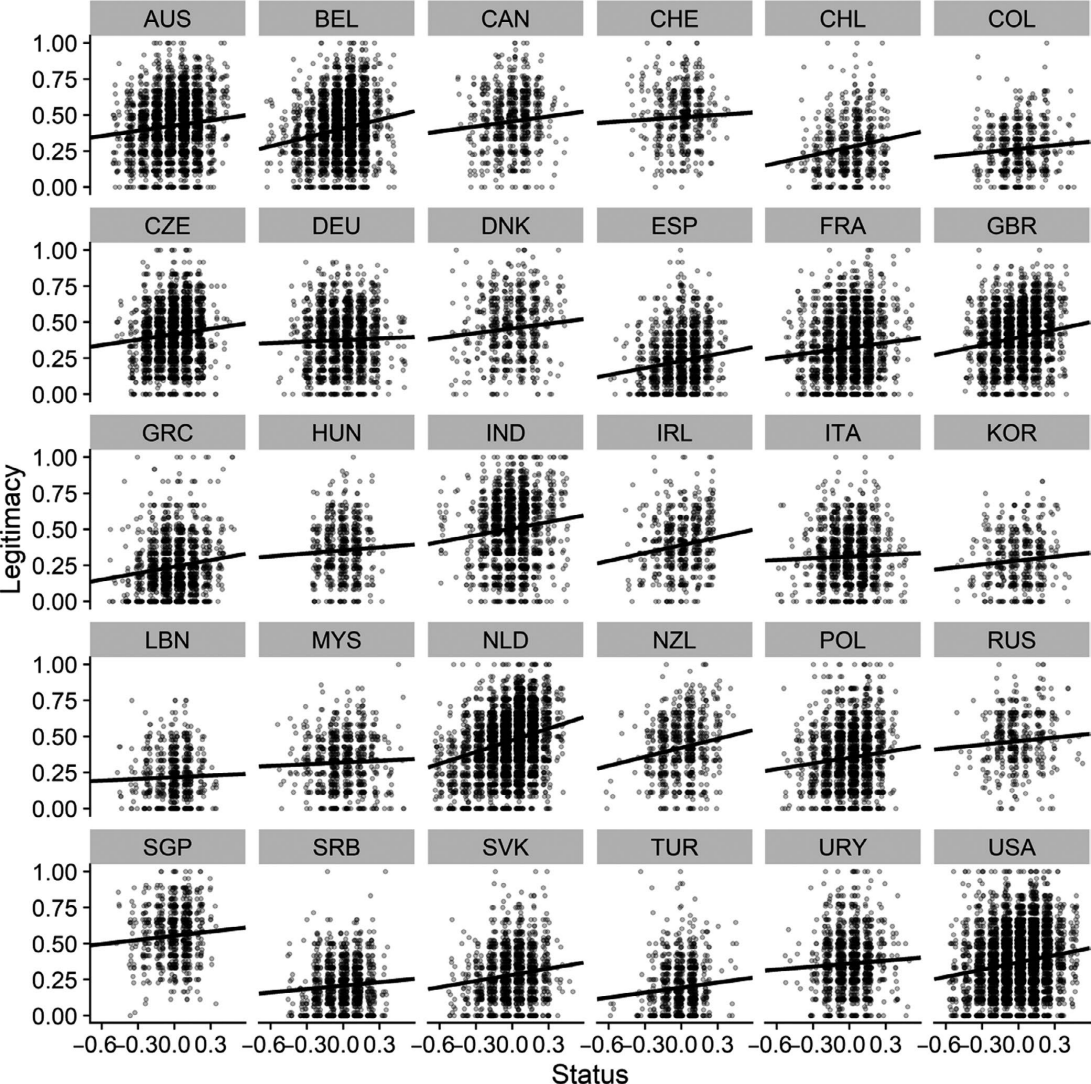
Estimated slopes of subjective status for each country from the multilevel model. Dots are randomly jittered horizontally to help show the data.

The effect of subjective status is not the same across all people in all situations. We re‐estimated the model with covariates and included random slopes at the country‐level for subjective status and sense of power. The estimated slope of subjective status on perceived legitimacy for each of the countries is in Figure 2. Although the effect of subjective status is always estimated to be positive when predicting perceived legitimacy, it varies in size. In the next section of the article, we consider moderators of the effects of subjective status.

### 3.2 Approach 1: Moderators of subjective status on perceived legitimacy

**FIGURE 3:**
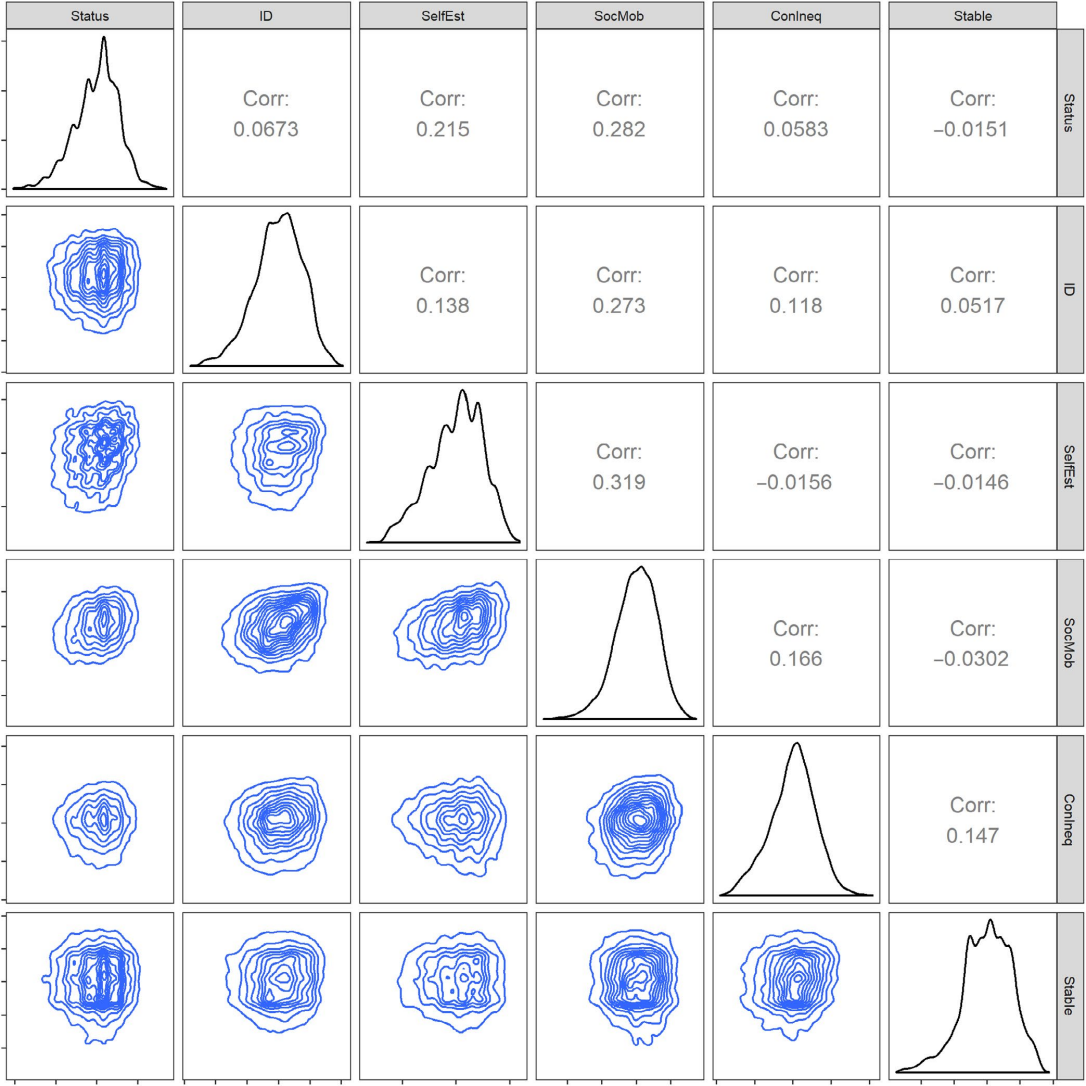
Correlations (above the diagonal) and density plots (below the diagonal) between primary predictor variables and the individual level variables. All variables were country‐mean centered before calculating the correlations. Density plots for each individual variable are on the diagonal. ID = Group identification; SelfEst = Self‐esteem; SocMob = Social mobility; ConInqu = Contribution to inequality; Stable = Stability of the status hierarchy [Colour figure can be viewed at wileyonlinelibrary.com]

We tested individual‐level moderators and country‐level moderators. Individual level moderators were country‐mean‐centered and included in separate models as fixed effects in the four‐level multilevel model used above (see Figure 3 for correlations between these measures).^11^We used the simr package in R (Green & MacLeod, 2016) to see how much power our model and sample size had to detect individual‐level interaction effects when *b* = .02, .05, .10, and .20. We used the model with the identification moderator as our base model for these analyses. These analyses showed that we had adequate power to detect moderation effects of at least .10 (i.e., a .10 difference in the unstandardized slope of status/power at the minimum and maximum of the moderator variable; power ≈12%, 46%, 98%, 100% respectively). We built the country‐level models using the same four‐level multilevel models, with the addition of the country‐level moderators and country‐level covariates.^12^We used the simr package in R (Green & MacLeod, 2016) to see how much power our model and sample size had to detect country‐level interaction effects for both subjective status and sense of power when *b* = .01, .02, .05, .10, and .20 We used the model with the meritocracy moderator as our base model for these analyses. These analyses showed that we had adequate power to detect country‐level moderation effects of at least .20 (power ≈6%, 10%, 45%, 87% respectively). All country‐level variables were grand‐mean‐centered, with the exception of the contrast codes for continents.^13^To check the robustness of our results to outliers at the country‐level (cf. Ullrich, & Schlüter, 2012), we visually inspected histograms of the country‐level predictors. There were clear outliers for both the Gini index and the measure of civil liberties. These models were run both with and without outliers. All models are in the figures summarizing results and the primary models discussed in the text include all data. In the country‐level models, we included random slopes for subjective status and sense of power.

To interpret the interactions, we used marginal effects plots created with interplot (Solt & Hu, 2015). This plots the marginal effect (sometimes called the simple effect) of the predictor variable for the entire range of the moderator variable. This helps us to understand the effect across the range of the moderator variables, rather than only focusing on points ±1 *SD* of the mean of the moderator variable (cf. Aiken & West, 1991). We also included a histogram of the moderator variable in each plot to illustrate how much of the sample is located at different levels of the moderator variable (cf. Hainmueller, Mummolo, & Xu, 2019).

#### 3.2.1 Moderators that reduce threat

We tested the hypotheses that are based on the assumption that group identification and self‐esteem reduce the experience of threat. If the hypotheses are supported, we should find positive interactions between the moderators and status, such that at high levels of the moderator variables the link between status and perceived legitimacy is positive and at low levels of the moderator variables the link is negative.

**FIGURE 4:**
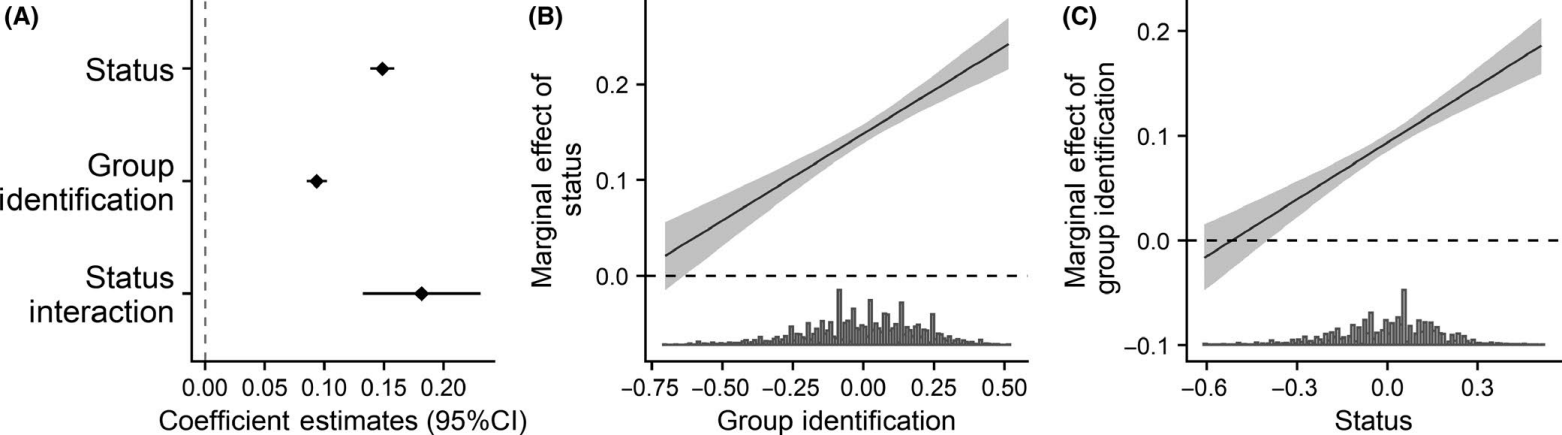
Panel A: Effects of subjective status and group identification on perceived legitimacy. Covariates are included in the model. Error bars are 95% confidence intervals. Panel B: Marginal effect of subjective status on perceived legitimacy (*y*‐axis) across the range of group identification (*x*‐axis). Panel C: Marginal effect of group identification on perceived legitimacy (*y*‐axis) across the range of subjective status (*x*‐axis). For both Panels B and C, the grey band around the slope is the 95% confidence interval. In all panels a null effect is highlighted with the dashed line

**FIGURE 5:**
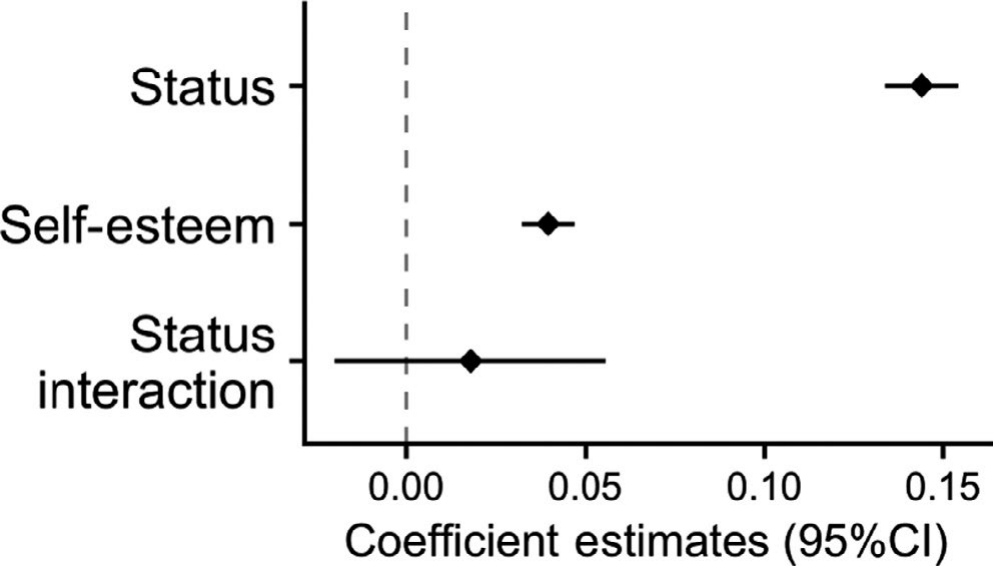
Effects of subjective status and self‐esteem on perceived legitimacy. Covariates are included in the model. Error bars are 95% confidence intervals. A null effect is highlighted with the dashed line

The results for group identification and self‐esteem are in Figures 4 and 5, respectively. One significant positive interaction emerged between identification and subjective status (Figure 4a). The positive marginal effect of subjective status is weaker at low levels of identification and non‐significant at the lowest levels of identification (Figure 4b). It is stronger and significant at high levels. This interaction is in the direction predicted by the hypothesis; however, the effect of subjective status is never significantly negative. Because this effect is not negative, it is only partially consistent with the full prediction that perceived legitimacy will be higher for low status than for high status groups among lower identifiers.

Self‐esteem, however, did not significantly interact with subjective status (Figure 5). In short, although group identification showed partial support for the hypotheses in the status domain, there was no support for the hypothesis for self‐esteem.

#### 3.2.2 Moderators that increase threat through dissonance

Inequality contribution, civil liberties, meritocracy, and inequality were all expected to increase the experience of threat through dissonance. If the hypotheses are supported, we should find negative interactions between the moderators and status, such that at high levels of the moderator variables the link between status and perceived legitimacy is negative and at low levels of the moderator variables the link is positive.

**FIGURE 6:**
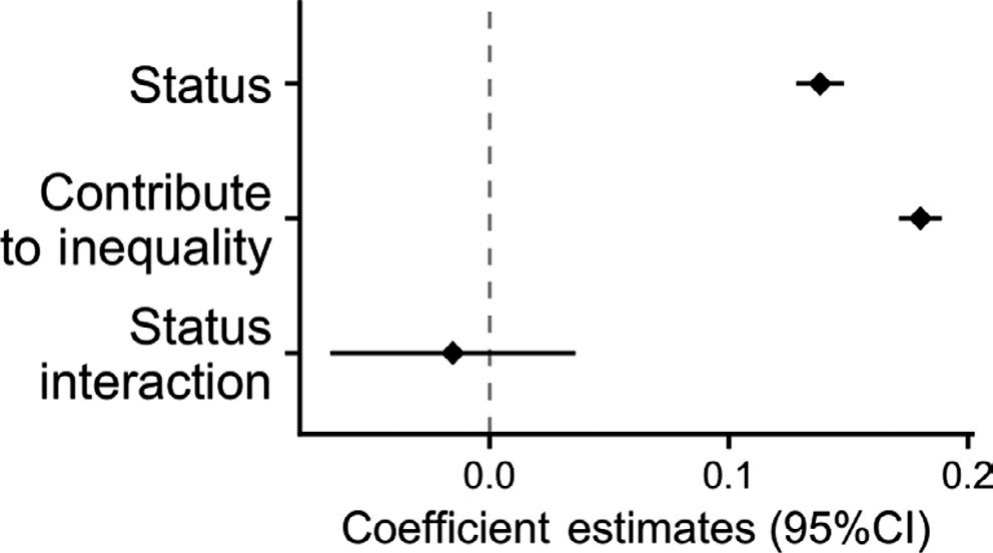
Effects of subjective status and contributing to inequality on perceived legitimacy. Covariates are included in the model. Error bars are 95% confidence intervals. A null effect is highlighted with the dashed line

**FIGURE 7:**
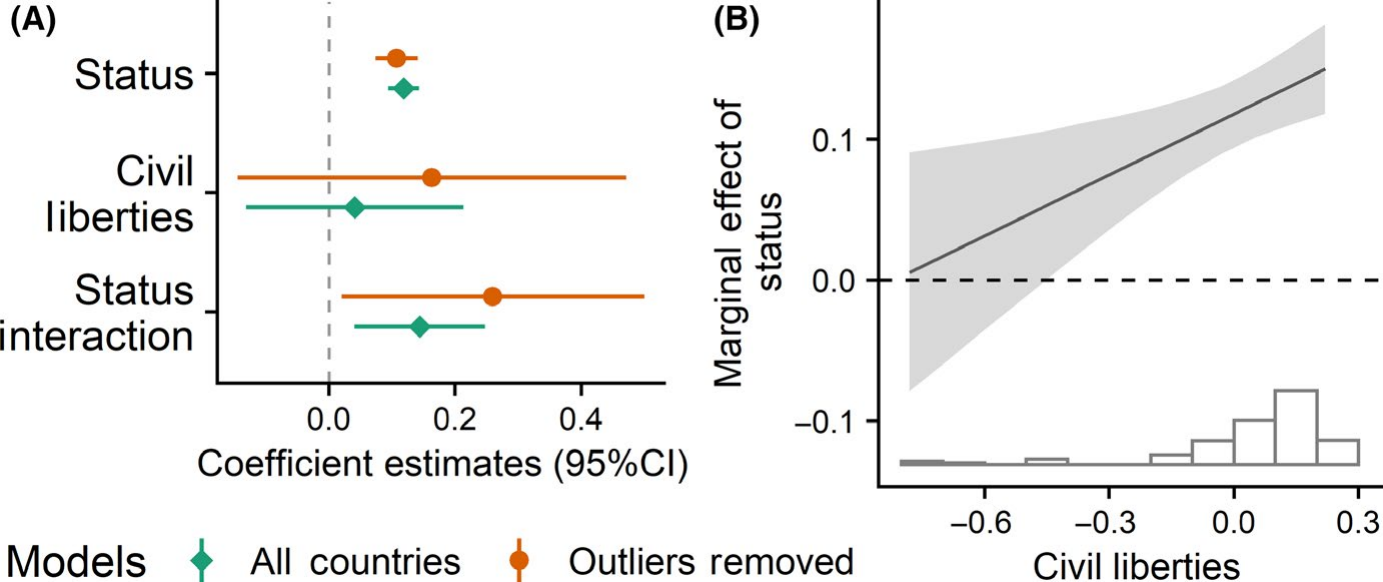
Panel A: Effects of subjective status and civil liberties on perceived legitimacy. Covariates are included in the model. Error bars are 95% confidence intervals. Panel B: Marginal effect of subjective status on perceived legitimacy (*y*‐axis) across the range of civil liberties (*x*‐axis). The grey band around the slope is the 95% confidence interval. In all panels a null effect is highlighted with the dashed line. See footnote 13 for outlier explanation [Colour figure can be viewed at wileyonlinelibrary.com]

**FIGURE 8:**
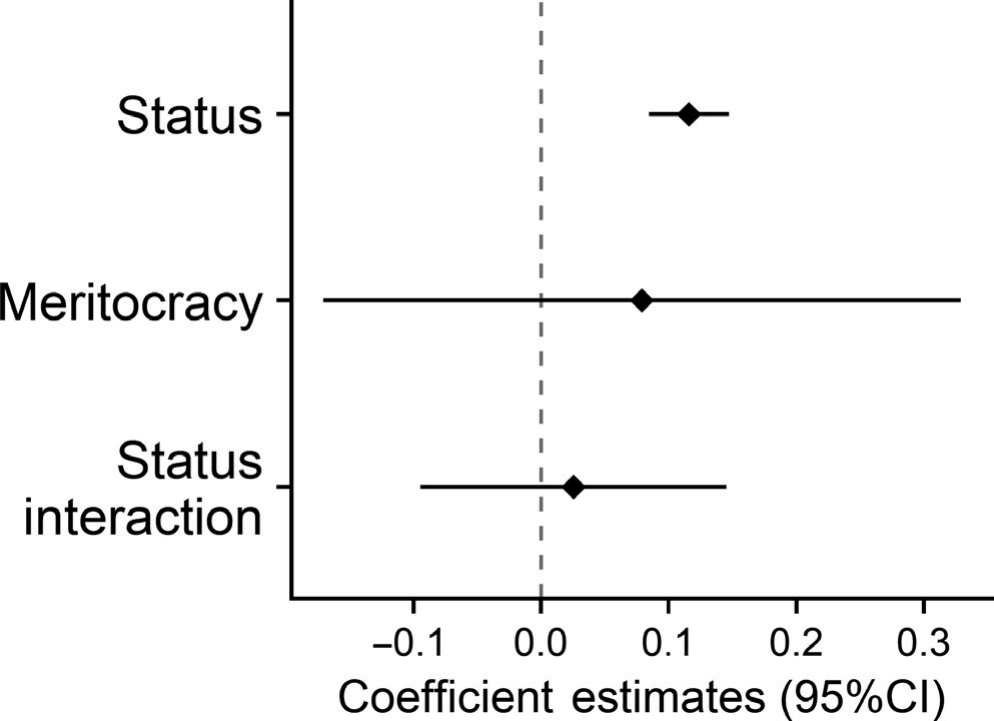
Effects of subjective status and meritocracy on perceived legitimacy. Covariates are included in the model. Error bars are 95% confidence intervals. A null effect is highlighted with the dashed line

**FIGURE 9:**
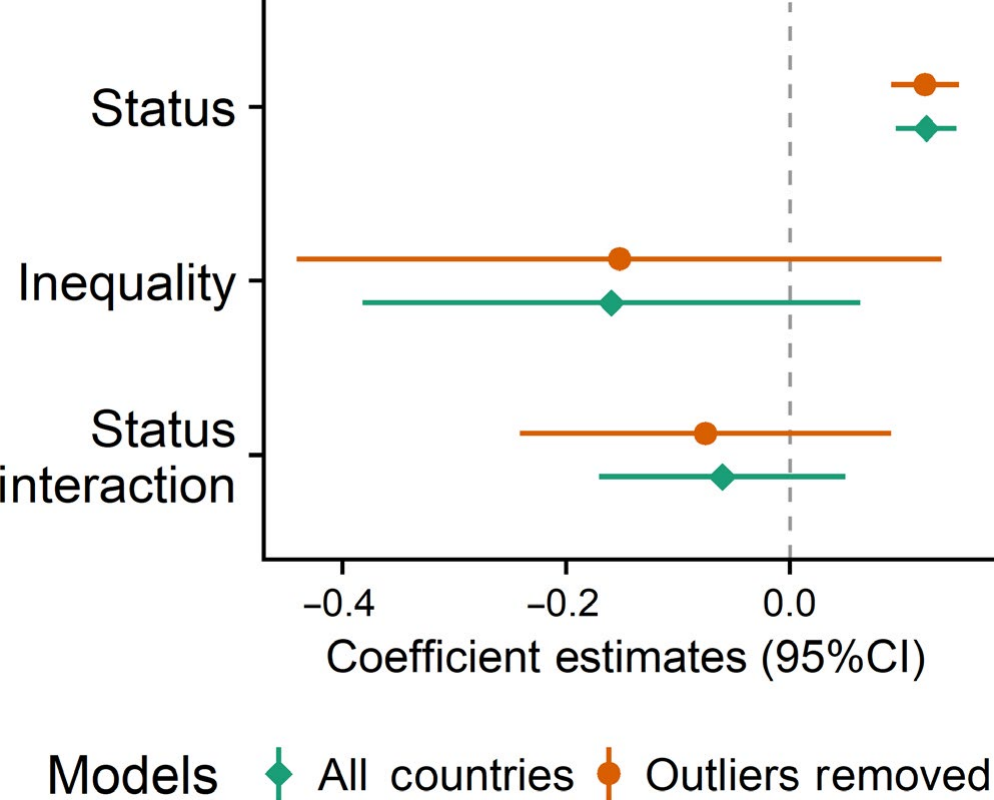
Effects of subjective status and inequality on perceived legitimacy. Covariates are included in the model. Error bars are 95% confidence intervals. A null effect is highlighted with the dashed line. See footnote 13 for outlier explanation [Colour figure can be viewed at wileyonlinelibrary.com]

There were no significant negative interactions between any of the four moderator variables expected to increase the experience of threat (Figures 6, 7, 8, 9). In the case of civil liberties, there was a positive interaction between civil liberties and subjective status. When probing the interaction, we found that the effect of subjective status (Figure 7b) was positive and *stronger* at high levels of civil liberties. At lower levels of civil liberties, the effect of subjective status was still positive, but weaker and non‐significant. This interaction is in the opposite direction predicted by the hypothesis. In short, in no cases did we find support for the hypotheses about moderators that could increase the experience of threat.

#### 3.2.3 Structural factors as moderators

The hypotheses suggested that inequality and perceived stability of the social system were structural factors that might moderate the association between status and perceived legitimacy. In contrast to the hypothesis tested in the prior paragraph, one hypothesis is that countries with high levels of inequality will have stronger, positive associations between status and perceived legitimacy because it makes status differences more salient. We did not find evidence for this (Figure 9).

**FIGURE 10:**
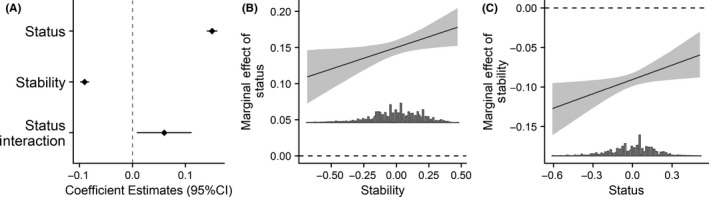
Panel A: Effects of subjective status and stability of the subjective status hierarchy on perceived legitimacy. Covariates are included in the model. Error bars are 95% confidence intervals. Panel B: Marginal effect of subjective status on perceived legitimacy (*y*‐axis) across the range of stability (*x*‐axis). Panel C: Marginal effect of stability on perceived legitimacy (*y*‐axis) across the range of subjective status (*x*‐axis). For Panels B and C the grey band around the slope is the 95% confidence interval. In all panels a null effect is highlighted with the dashed line

For stability, the hypothesis predicted that the link between status and perceived legitimacy would be negative when perceived stability was high compared to low. This hypothesis would be supported with a negative interaction effect. This effect did not emerge (Figure 10a). Instead, we found the opposite interaction effect (Figure 10b). Contrary to the hypothesis, the link between status and perceived legitimacy is strong and positive when stability is high and it is weaker or non‐significant when stability is low.

In short, we do not find evidence that inequality may exacerbate status‐based conflict. We also find that stability is a significant moderator, but in the opposite direction from that expected. It indicates that stability is interpreted differently in the context of real‐life social inequality (see also Verkuyten & Reijerse, 2008), compared to stability that is manipulated in groups created in the lab (e.g., Ellemers et al., 1990). A possible interpretation of the pattern found in this study could be that the perception that social inequality will not change might be an additional reason for judging that inequality is unacceptable (assuming people perceive there to be inequality). At the same time, we note that the reliability of our stability measure could be improved.

### 3.3 Approach 2: Predictors of perceived legitimacy for people with low levels of subjective status

We tested the hypotheses about the predictors of perceived legitimacy for people with low levels of subjective status using the same models described and presented above. To assess whether there was support for the hypotheses, we first examined the main effects of the predictors on perceived legitimacy. This main effect analysis tells us whether there is an average effect across the sample. When there is a significant interaction between the predictor and status, we examine whether the predictor still has the expected effect (e.g., a positive effect of social mobility) for people with lower levels of status (regardless of the pattern for those higher in status). This will tell us whether the effect of the predictor is specific to people with low social status or sense of power.

**FIGURE 11:**
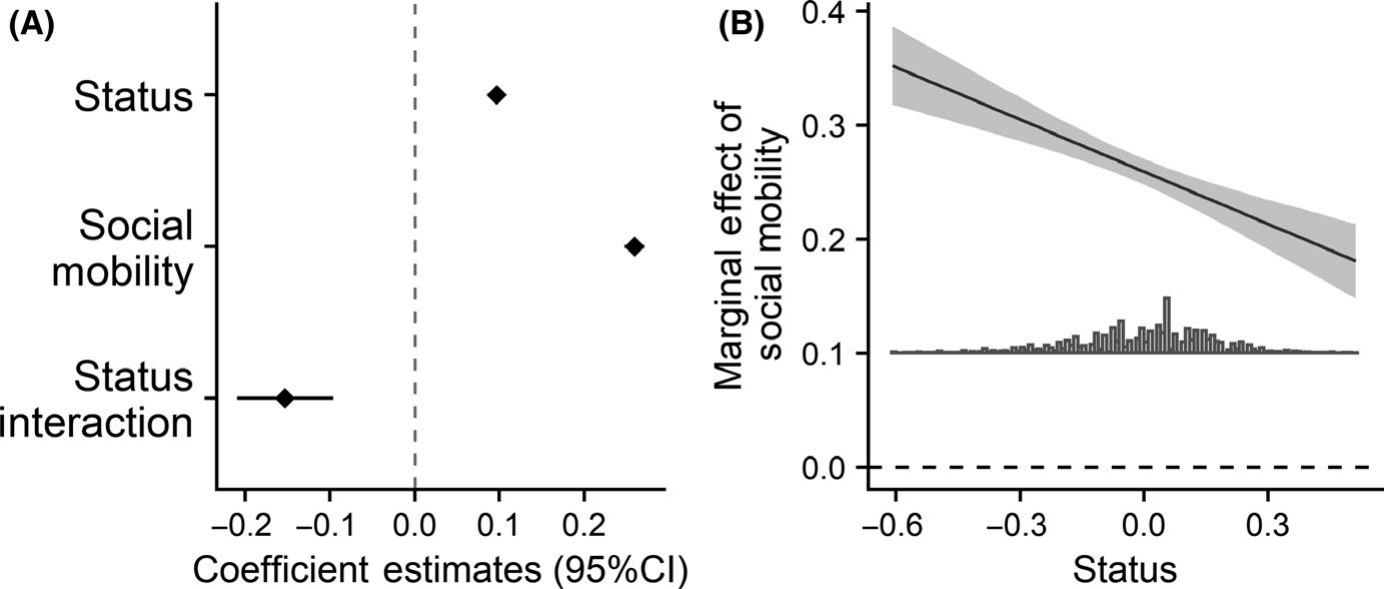
Panel A: Effects of subjective status and social mobility on perceived legitimacy. Covariates are included in the model. Error bars are 95% confidence intervals. Panel B: Marginal effect of social mobility on perceived legitimacy (*y*‐axis) across the range of subjective status (*x*‐axis). For Panel B, the grey band around the slope is the 95% confidence interval. In all panels a null effect is highlighted with the dashed line

The hypotheses predicted that people would see the system as more legitimate when it fulfilled a social mobility or stability related goal. For social mobility, there was clear support for the hypothesis. There was a large positive effect of social mobility, such that higher levels of perceived social mobility were associated with higher levels of perceived legitimacy (Figure 11a). This positive effect was moderated by subjective status (Figure 11a), suggesting that the positive effect is variable across levels of subjective status. At low levels of subjective status (Figure 11b), perceived social mobility remained a significant positive predictor of perceived legitimacy. This is consistent with the hypothesis.

For stability, results were not consistent with the hypothesis. There was a negative effect of stability on perceived legitimacy (Figure 10a). This negative effect was moderated by subjective status (Figure 10a); however, at low levels of subjective status (Figure 10c) the effect of stability remained negative and significant.

In short, when people see social mobility as a possibility, people with low subjective status see the system as more legitimate; however, perceiving the system as more stable is associated with less legitimacy at lower levels of status, which is inconsistent with the hypothesis.

We also made the prediction that when personal or group interests are fulfilled, perceived legitimacy of the system is less necessary. The hypotheses predicted that identification and self‐esteem would be negatively associated with levels of perceived legitimacy for people with low levels of subjective status.

Inconsistent with hypotheses, there are significant positive effects of identification (Figure 4a) and self‐esteem (Figure 5). To see whether this was consistent across people with low subjective status, we examined the interaction effects. The positive effect of self‐esteem was not moderated by subjective status (Figure 5). The positive effect of group identification was moderated by subjective status (Figure 4a). Here, we find that the effect of identification is not different from zero for people who are very low status (Figure 4c). Although this does not confirm the hypothesis, it is also not contrary to it.

## 4 GENERAL DISCUSSION

Debates around status and perceptions of legitimacy in psychology are characterized by mixed findings and different theoretical foci from different research groups. Consistent with some past work (e.g., Brandt, 2013; Caricati, 2017; Caricati & Lorenzi‐Cioldi, 2012; Kraus & Callaghan, 2014; Vargas‐Salfate et al., 2018; Zimmerman & Reyna, 2013), but inconsistent with the status‐legitimacy hypothesis (e.g., Henry & Saul, 2006; Jost et al., 2003), we found that subjective status is positively associated with perceived legitimacy. However, this is not our primary contribution. We sought to advance this debate beyond straightforward main effects by taking two approaches.

### 4.1 Approach 1: Moderator of the association between subjective status and perceived legitimacy

Approach 1 examined how constructs that reduce threat, constructs that increase threat, and perceived structural factors may be moderators of the association between status and perceived legitimacy. We tested eight specific moderator hypotheses and found partial support for one: the identification‐moderation hypothesis (see Table 4). That is, the most common result was no clear support for the hypotheses. Sometimes the lack of support was due to non‐significant interactions. The sensitivity analyses reported in footnotes 9, 11, and 12 indicate that we have substantial power to detect main effects and interactions at the individual level. For country‐level interaction effects, we only had sufficient power to detect large effects. In the cases of null results, studies with even greater statistical power may find evidence in support of these hypotheses, in opposition to these hypotheses, or for a null effect (time will tell). In many other cases, the lack of support was due to a significant interaction that was the *opposite* of the prediction (e.g., stability‐moderator hypothesis). In these cases (highlighted with ✗^op^ in Table 4), it seems less plausible that the issue was a lack of statistical power. The predictive power of the hypotheses for moderators was essentially nil.

**TABLE 4:** Summary of moderation hypotheses (Approach 1) and whether they were supported

Moderator hypothesis	
*Reduce threat*	
Identification‐moderation hypothesis	✓
Self‐esteem‐moderation hypothesis	✗
*Increase threat through dissonance*	
Inequality contribution‐moderation hypothesis	✗
Civil liberties hypothesis	✗^op^
Meritocracy hypothesis	✗
SJT inequality hypothesis	✗
*Structural factors that affect threat*	
SIT inequality hypothesis	✗
Stability‐moderator hypothesis	✗^op^

Note

✓ = indicates support and partial support for the hypothesis, ✗ = indicates no support for the hypothesis. ✗^op^ = indicates a significant interaction effect in the opposite direction.

Abbreviations: SIT, Social identity theory; SJT, System justification theory.

### 4.2 Approach 2: Predictors of perceived legitimacy for people with *lower* levels of subjective status

Approach 2 examined the predictors of perceived legitimacy for people with low subjective status. We tested four specific hypotheses about these predictors and found support for one: the social mobility‐legitimacy hypothesis (see Table 5). Most of the predictions were not confirmed and, in most cases, results were opposite to the predicted direction. As one example, the identification‐legitimacy hypothesis predicted a negative association for members of low status/power groups. Yet, we found the opposite. Some members of our team have started to think through this type of effect (and have published some findings consistent with the data in this manuscript). As explained earlier, they have proposed that among people with low subjective status, identification will be positively associated with system legitimacy, especially when the system can be used as a vehicle for improvements to group status in the long term (Owuamalam, Rubin, & Spears, 2018). Our data lendsome credence to this hypothesis.

**TABLE 5:** Summary of predictor hypotheses (Approach 2) and whether they were supported

Predictor hypothesis	
Social Mobility‐Legitimacy Hypothesis	✓
Stability‐Legitimacy Hypothesis	✗^op^
Identification‐legitimacy hypothesis	✗
Self‐esteem legitimacy hypothesis	✗^op^

Note

✓ = indicates support and partial support for the hypothesis, ✗ = indicates no support for the hypothesis. ✗^op^ = indications a significant interaction effect in the opposite direction.

Although almost never in a direction supportive of the hypotheses (see Table 5), we find that group identification, self‐esteem, and beliefs in social mobility are all associated with greater perceived legitimacy among people with low subjective status. In this way, we helped fulfill Jost’s (2017) call to better understand what leads people who are oppressed to uphold the system.

### 4.3 Strengths, limitations, and future directions

We used a design that leverages larger samples from multiple countries to understand how subjective status is associated with perceived legitimacy and related constructs. This helps us avoid low statistical power (Sedlmeier & Gigerenzer, 1989) and uses less‐WEIRD sampling (Henrich, Heine, & Norenzayan, 2010). That said, hardly any of the samples were representative, and we were not able to recruit participants from all the regions of the globe. Most notably, we were unable to secure data from the continent of Africa, and Asia is underrepresented. However, given the consistency of our results with those of researchers who have used data from even more diverse and representative samples (Brandt, 2013; Caricati, 2017; Caricati & Lorenzi‐Cioldi, 2012; Vargas‐Salfate et al., 2018), we believe that our data provide a reasonable approximation at this time for the regions that we do cover. A related limitation is that although we report the range of Cronbach's *α* and *r* for all scales across countries, we have not formally tested measurement invariance (He & van de Vijver, 2012). It is encouraging that some of the scales we use have been shown to be invariant across at least some countries (e.g., Davidov & Coromina, 2013). Clearly, more work is needed on the social psychology of perceived legitimacy and its development in understudied regions; this future work will surely have both practical and theoretical benefits.

Many of our samples are student samples. This allowed us to use our resources efficiently, but necessarily implies that the samples are younger and better educated than the general population. Although studies on similar topics (and social psychology more broadly) have relied on student and non‐representative samples, it is always possible that samples with more objectively and subjectively low status people, or that deliberately recruit people from disadvantaged groups (e.g., homeless shelters, soup kitchens) would reveal different results. That being said, 34% of our sample self‐reported a subjective social status below the midpoint of the scale, indicating that our sample cannot easily be dismissed as a sample consisting only of people who seem themselves as high status. Moreover, our samples included representative (sample NLD3) and community samples similar to those which have been used to study low social status in other work (e.g., MTurk in sample USA1 was used in Plantinga, Krijnen, Zeelenberg, & Breugelmans, 2018; Shah, Shafir, & Mullainathan, 2015). Additionally, our student samples are from a diverse array of university types, including public and private universities and community colleges serving a diverse range of students. Lastly, the main effects of status are similar to studies using representative samples (e.g., Brandt, 2013), suggesting that our findings are consistent with data using other sampling techniques.

### 4.4 Where to now?

Our project provides scholars with additional evidence that they can use to inform the direction of research on status and legitimacy.^14^See Appendix S1 for data that can inform scholars interested in people's sense of power. This additional evidence comes in two forms. *First*, these hypotheses were inspired by both social identity and system justification theories and primarily made inaccurate predictions in our study. This may be because the prediction was wrong, or because some auxiliary assumptions did not hold in our particular samples and measures. For example, nearly every moderator predicted to reduce feelings of threat or increase feelings of threat through dissonance were not supported. This may mean that these moderators are not associated with threat as expected (auxiliary assumption did not hold), or that threat is not a key mechanism linking status to legitimacy (theoretical prediction was wrong). Learning from theoretical failures and inaccurate predictions like these can often be more informative than learning from theoretical successes (Ferguson & Heene, 2012; Popper, 1959).

It is also plausible that some of the inaccurate predictions (and perhaps most of them) can be addressed by increasing the specificity of the theories and how theoretical concepts are translated into concrete operationalizations. For example, social identity theory has traditionally treated legitimacy as a moderator and used outcome measures such as group evaluations, allocation, and social change strategies. Here, we tried to extend the theory's predictions to legitimacy, but did not find consistent support. This may suggest that social identity theory's predictions about social change strategies do not extend to legitimacy in a straightforward way, which may also help to explain the limited support for predictions derived from social identity theory. Recent work has therefore investigated how a social identity perspective *can* be better applied to predict system legitimacy (Owuamalam et al., 2019).

*Second*, our data provide descriptive information about the precisely estimated associations between a large number of socially and politically relevant variables. That is, not only did our study fail to confirm a number of hypotheses about status and legitimacy, but it also provides the relevant associations and non‐associations that relevant theories need to explain. For example, despite expectations of both perspectives, a negative association between status and legitimacy was not found at low levels of identification. Theories need to account for this pattern of results. Similarly, perceived stability was associated with lower levels of legitimacy, despite expectations. Updates will need to account for this pattern of results. Although post‐hoc explanations are possible, we hope that theorists can build clearly specified models that can be used to explain the current data and to make new, falsifiable predictions (e.g., Muthukrishna & Henrich, 2019).

### 4.5 Conclusion

We started this project because we noted that there is evidence both consistent and inconsistent with the status‐legitimacy hypothesis. Our study found very little support for moderator hypotheses across 30 countries and several moderator variables. To the extent that the phenomenon predicted by the status‐legitimacy hypothesis exists, this appears to be quite rare. That does not mean that people with low subjective status never see the system as relatively legitimate. Our data also shed light on when this is most likely to be the case. We find that group identification, self‐esteem, and beliefs in social mobility are all associated with perceived legitimacy among people who are low status (as well as those with high status). We hope that our findings inspire scholars interested in this domain to pursue questions related to status and legitimacy to enrich our understanding of these constructs around the world.

## CONFLICTS OF INTEREST

There are no conflicts of interest to report.

## TRANSPARENCY STATEMENT

Data, code, and materials are available at the following link: https://osf.io/5uxc7/.

## Supporting information

Appendix S1
