# Supplementary material for: Subjective status and perceived legitimacy across countries
Source: Eur J Soc Psychol. 2020 Jun 30;50(5):921–42. doi: 10.1002/ejsp.2694 (PMC7507836; doi:10.1002/ejsp.2694)
Supplement: Supplementary file 1 — Appendix S1 [file EJSP-50-921-s001.docx]

**Supplemental Materials for**

**Subjective Status and Perceived Legitimacy across Countries**

This document contains supplementary information, analyses, and summaries.

# Pre-registration

We preregistered the research questions here: <https://osf.io/tgs4a/?view_only=19d694d4b76f4805b7befd39aab595e6>. We also intended to report results for perceptions of admiration and contempt for high status groups. However, in drafting the manuscript it became obvious that these additional dependent variables substantially decreased the readability of the paper. Admiration and contempt also tended to be weakly correlated with the other measures of perceived legitimacy (mean *r*’s = .19) and with each other (*r* = -.008). Analyses with these variables are included below. The preregistration includes the original research questions and hypotheses; however, please also note that some hypotheses were added or removed during the drafting of the manuscript to address various concerns (e.g., other plausible hypotheses, increase readability). This preregistration was imperfect.

# Complete list of measures and manipulations

| respnr | respondent number |
| --- | --- |
| age | Age: |
| sex | What is your gender? |
| country | three letter country code |
| labs | lab which collected the data |
| sample | community vs student sample |
| paper | online vs paper |
| BLOCK_1 | used for creating conditions (see below) |
| BLOCK_2 | used for creating conditions (see below) |
| power_1 | I can get him/her/them to listen to what I say. |
| power_2 | My wishes do not carry much weight. |
| power_3 | I can get him/her/them to do what I want. |
| power_4 | Even if I voice them, my views have little sway. |
| power_5 | I think I have a great deal of power. |
| power_6 | My ideas and opinions are often ignored. |
| power_7 | Even when I try, I am not able to get my way. |
| power_8 | If I want to, I get to make the decisions |
| status | Think of this ladder as representing where people stand in the United States.   At the top of the ladder are people who are the best off - those who have the most money, the most education, and the most respected jobs. At the bottom are the people who are the worst off - who have the least money, least education, and the least respected jobs or no job. The higher up you are on this ladder, the closer you are to the people at the very top; the lower you are, the closer you are to the people at the very bottom. Please choose the number of the rung of the ladder where you think you stand at this time in your life, relatively to other people in the United States. |
| selfeff_1 | I can learn almost anything if I set my mind to it. |
| selfeff_2 | I can usually achieve what I want if I work hard for it. |
| selfeff_3 | Almost anything is possible for me if I really want it. |
| selfeff_4 | I have high self-esteem. |
| poleff_1 | The average citizen can have an influence on government decisions. |
| poleff_2 | By taking an active part in political and social affairs we, the people, can control world events. |
| poleff_3 | It is difficult for us to have much control over the things politicians do in office. |
| moveup_1 | I am motivated to climb up the social ladder. |
| moveup_2 | I am able to climb up the social ladder |
| moveup_3 | In general, people can easily get ahead in society. |
| moveup_4 | In general, people can climb the social ladder and be successful |
| esteemid_1 | People with a similar background and social class to my own can easily get ahead in society. |
| esteemid_2 | It is easy for people with a similar background and social class to my own to climb the social ladder and be successful. |
| esteemid_3 | I think that people from my social class have a lot to be proud of |
| esteemid_4 | It is pleasant to be a member of my social class |
| esteemid_5 | In general, others respect people from my social class |
| esteemid_6 | In general, others think that people from my social class are unworthy |
| esteemid_7 | I identify with people from my social class |
| esteemid_8 | I feel solidarity with my social class |
| esteemid_9 | My social class is an important part of how I see myself |
| classfocus_1 | When I think of my social class, I think first and foremost about who I feel at home with. |
| classfocus_2 | When I think of my social class, I think about people who have shared interests. |
| legstab_1 | Differences in power and status between groups in the United States are… ...illegitimate. |
| legstab_2 | Differences in power and status between groups in the United States are… ...unfair. |
| legstab_3 | Differences in power and status between groups in the United States are… ...difficult to change. |
| legstab_4 | Differences in power and status between groups in the United States are… ...will remain stable over time. |
| coninequ_1 | I contribute to keeping society the way it is |
| coninequ_2 | I contribute to maintaining the current social hierarchy |
| coninequ_3 | I could do more to change differences in power and status between groups in society |
| coninequ_4 | I don't do anything to change the current differences in power and status in society |
| coninequ_5 | I am not trying to change the current differences in power and status in society |
| sj_1 | In general, I find society to be fair. |
| sj_2 | In general, the United States' political system operates as it should. |
| sj_3 | Society needs to be radically restructured. |
| sj_4 | The United States is the best country in the world to live in. |
| sj_5 | Most policies serve the greater good. |
| sj_6 | Everyone has a fair shot at wealth and happiness. |
| sj_7 | Our society is getting worse every year. |
| sj_8 | Society is set up so that people usually get what they deserve. |
| emos_1 | How much admiration do you feel towards people at the very top of the ladder? |
| emos_2 | How much admiration do you feel towards people at the very bottom of the ladder? |
| emos_3 | How much contempt do you feel towards people at the very top of the ladder? |
| emos_4 | How much contempt do you feel towards people at the very bottom of the ladder? |
| con_1 | The armed forces |
| con_2 | The police |
| con_3 | The courts |
| con_4 | The government of the country |
| con_5 | Congress |
| con_6 | Major companies |
| con_7 | Banks and financial institutions |
| trust_1 | How much of the time do you think you can trust the government to do what is right? |
| trust_2 | Would you say the government is pretty much run by a few big interests looking out for themselves or that it is run for the benefit of all the people? |
| trust_3 | Do you think that people in the government waste a lot of money we pay in taxes, waste some of it, or don’t waste very much of it? |
| trust_4 | Do you think that quite a few of the people running the government are crooked, not very many are, or do you think hardly any of them are crooked? |
| momedu | What is the highest level of education completed by your mother? |
| momedu_TEXT | Free text from "other" choice. Removed from publicly shared data |
| dadedu | What is the highest level of education completed by your father? |
| dadedu_TEXT | Free text from "other" choice. Removed from publicly shared data |
| momdadimm | Were your mother or father an immigrant to the United States? |
| imm | Were you born in the United States, or are you an immigrant? |
| lived | How long have you lived in the United States |
| empstat | Which of the following describes best what you have been doing for the last two weeks? |
| empstat_TEXT | Free text from "other" choice. Removed from publicly shared data |
| youedu | What is the highest level of education that you have completed? |
| youedu_TEXT | Free text from "other" choice. Removed from publicly shared data |
| curredu | Which study are you currently enrolled for? |
| curredu_TEXT | Free text from "other" choice. Removed from publicly shared data |
| ethnic | What is your ethnicity? Check all that apply. Removed from publicly shared data |
| ethnic_1 | What is your ethnicity? Check all that apply. Removed from publicly shared data |
| ethnic_2 | What is your ethnicity? Check all that apply. Removed from publicly shared data |
| ethnic_3 | What is your ethnicity? Check all that apply. Removed from publicly shared data |
| ethnic_4 | What is your ethnicity? Check all that apply. Removed from publicly shared data |
| ethnic_5 | What is your ethnicity? Check all that apply. Removed from publicly shared data |
| ethnic_6 | What is your ethnicity? Check all that apply. Removed from publicly shared data |
| ethnic_7 | What is your ethnicity? Check all that apply. Removed from publicly shared data |
| ethnic_8 | What is your ethnicity? Check all that apply. Removed from publicly shared data |
| ethnic_9 | What is your ethnicity? Check all that apply. Removed from publicly shared data |
| ethnic_10 | What is your ethnicity? Check all that apply. Removed from publicly shared data |
| ethnic_11 | What is your ethnicity? Check all that apply. Removed from publicly shared data |
| ethnic_TEXT | Free text from "other" choice. Removed from publicly shared data |
| labs_2 | old labs text before combination |
| condition | Experimental conditions (when included) |
| momedurc | Mother's education ISCED standardized |
| dadedurc | Father's education ISCED standardized |
| youedurc | Participant's education ISCED standardized |

# Means for each country on the individual-level variables

| **Country** | **Status** | **Power** | **ID** | **SelfEst** | **SocMob** | **ConIneq** | **Stable** | **SJ** | **Trust** | **Confid** | **Legit** |
| --- | --- | --- | --- | --- | --- | --- | --- | --- | --- | --- | --- |
| aus | 6.29 | 0.91 | 0.39 | 3.36 | 0.83 | 0.13 | 0.94 | -0.19 | 0.15 | 1.54 | -0.65 |
| bel | 6.46 | 1.06 | 0.30 | 3.08 | 0.98 | 0.01 | 0.65 | -0.36 | 0.27 | 1.44 | -0.78 |
| can | 6.02 | 0.82 | 0.34 | 3.42 | 0.79 | 0.21 | 0.65 | 0.08 | 0.29 | 1.64 | -0.35 |
| che | 6.72 | 0.99 | -0.09 | 2.75 | 0.84 | -0.13 | 0.68 | -0.02 | 0.51 | 1.67 | 0.04 |
| chl | 7.42 | 1.26 | 0.13 | 3.54 | 0.50 | -0.83 | 1.03 | -1.38 | -0.19 | 1.12 | -1.60 |
| col | 5.06 | 0.31 | 0.41 | 5.01 | 0.94 | -0.39 | 0.96 | -1.33 | -0.47 | 1.06 | -1.30 |
| cze | 6.07 | 1.04 | 0.49 | 2.99 | 0.91 | 0.05 | 0.52 | -0.56 | -0.03 | 1.38 | -0.29 |
| deu | 5.67 | 1.06 | 0.73 | 3.58 | 0.86 | -0.11 | 0.95 | -0.21 | 0.18 | 1.24 | -1.27 |
| dnk | 6.36 | 1.27 | 0.65 | 3.69 | 1.05 | -0.31 | 0.70 | 0.05 | 0.44 | 1.69 | -0.73 |
| esp | 6.00 | 0.15 | 0.71 | 3.68 | 0.41 | -0.66 | 0.92 | -1.57 | -0.53 | 0.95 | -1.47 |
| fra | 5.59 | 0.72 | 0.20 | 2.45 | 0.46 | -0.31 | 0.91 | -1.08 | -0.35 | 1.40 | -1.20 |
| gbr | 5.70 | 0.69 | 0.33 | 2.78 | 0.74 | 0.02 | 1.00 | -0.50 | 0.06 | 1.51 | -0.86 |
| grc | 5.60 | 0.77 | 0.70 | 3.91 | 0.41 | -0.75 | 1.01 | -1.74 | -0.65 | 0.80 | -1.21 |
| hun | 6.01 | 1.18 | 0.44 | 3.14 | 0.69 | -0.69 | 1.29 | -0.96 | -0.18 | 1.27 | -0.35 |
| ind | 6.16 | 0.77 | 1.22 | 4.68 | 1.40 | 0.42 | 0.87 | 0.42 | 0.35 | 1.91 | -0.61 |
| irl | 5.88 | 0.85 | 0.66 | 3.41 | 1.05 | -0.03 | 0.81 | -0.43 | 0.11 | 1.33 | -0.56 |
| ita | 6.08 | 1.24 | 0.53 | 4.25 | 0.57 | -0.56 | 0.95 | -1.07 | -0.28 | 1.23 | -1.04 |
| kor | 5.34 | 0.95 | 0.11 | 3.68 | 0.24 | -0.64 | 1.26 | -1.37 | -0.54 | 1.06 | -0.72 |
| lbn | 7.02 | 0.94 | 0.42 | 3.87 | 0.93 | -0.19 | 1.11 | -1.67 | -0.72 | 1.02 | -1.58 |
| mys | 5.69 | 0.53 | 0.59 | 3.68 | 1.12 | -0.16 | 0.53 | -0.68 | -0.43 | 1.20 | -1.02 |
| nld | 6.47 | 0.93 | 0.51 | 3.43 | 0.95 | 0.32 | 0.78 | 0.03 | 0.52 | 1.53 | -0.66 |
| nzl | 6.36 | 0.84 | 0.42 | 3.42 | 0.82 | -0.11 | 0.66 | -0.19 | 0.22 | 1.57 | -0.72 |
| pol | 5.72 | 1.06 | 0.51 | 3.32 | 0.96 | -0.35 | 0.74 | -0.91 | -0.42 | 1.27 | -0.40 |
| rus | 5.40 | 1.37 | 0.95 | 3.06 | 1.64 | 0.17 | 1.02 | -0.35 | 0.65 | 1.51 | 0.02 |
| sgp | 6.10 | 0.71 | 0.49 | 3.01 | 0.88 | 0.52 | 1.01 | 0.44 | 1.07 | 1.96 | -0.24 |
| srb | 5.96 | 1.06 | 0.17 | 3.89 | 0.52 | -0.67 | 0.81 | -1.96 | -0.60 | 0.80 | -1.59 |
| svk | 5.50 | 0.90 | 0.76 | 3.13 | 0.49 | -0.04 | 1.05 | -1.21 | -0.38 | 0.99 | -1.22 |
| tur | 6.16 | 1.50 | 0.67 | 4.57 | 0.83 | -0.65 | 0.17 | -2.09 | -0.54 | 0.83 | -1.63 |
| ury | 5.63 | 0.39 | 1.01 | 4.82 | 1.03 | -0.50 | 0.64 | -0.91 | 0.11 | 1.16 | -0.53 |
| usa | 5.87 | 0.94 | 0.53 | 3.76 | 0.76 | -0.26 | 0.79 | -0.69 | -0.16 | 1.37 | -0.76 |

# Country-level variable histograms

To check for outliers for variables at the country-level, we plotted and visually inspected the four country-level moderators (Figure S1). Countries scoring over .3 for the Gini Index and lower than -.3 for Civil Liberties were removed for the analyses excluding outliers.


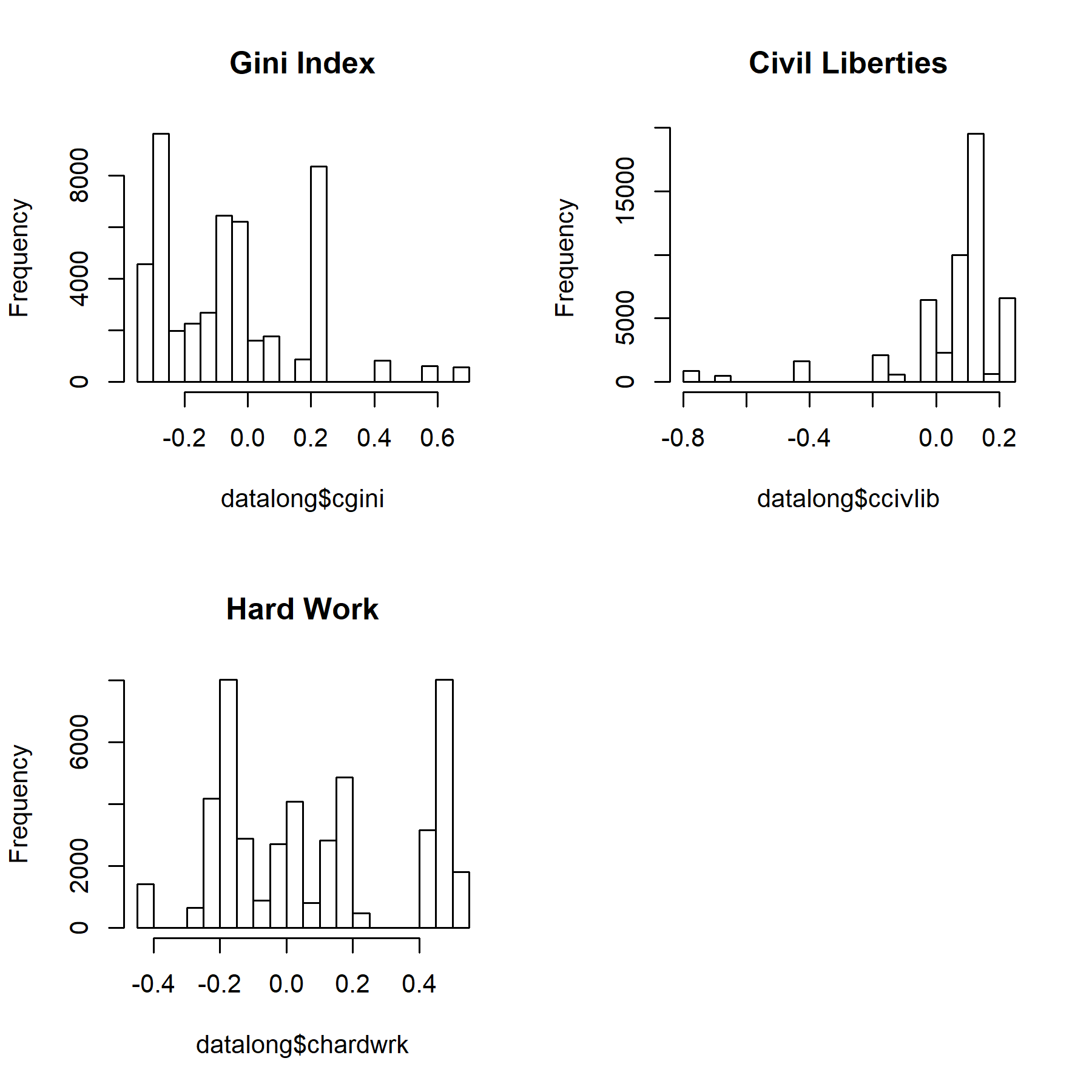


Figure S1. *Histograms of country-level moderators*

# Sense of Power Results

In the initial submission of this manuscript to the *European Journal of Social Psychology*, we included personal sense of power as a second primary predictor. Our intention was to report tests of the power-legitimacy hypothesis, which predicts that people with lower power are more likely to see the system as legitimate. The editor and a reviewer suggested removing these analyses to streamline the paper. Moreover, although personal sense of power was used in a past test of the power-legitimacy hypothesis, it may not effectively match a more contextual sense of power required for system legitimacy. Therefore, we have moved the results here. Note: the figures with the results below are the figures from the initial submission of this manuscript. Moreover, the models in these figures are the same exact models as the models in the main text. In the main text, we merely removed the focus on sense of power, but did not remove sense of power from the models (see description of models in main text).

We measured sense of power with the personal feelings of power scale (Anderson, John, & Keltner, 2012). This scale consists of eight items, prefaced by the phrase “In my relationships with others…” that are answered on a scale ranging from -3 = disagree strongly to 3 = agree strongly. This includes items such as “I can get him/her/them to listen to what I say,” and “Even if I voice them, my views have little sway” (reverse scored). It had good reliability (α = .76), captures the low levels of control thought to be the mechanism for the power-legitimacy hypothesis, and was used in one prior study on sense of power and system legitimacy conducted among university students (van der Toorn et al., 2015, Study 2). Responses in our sample were above the midpoint (M = 0.90, SD = 0.85), but spanned the entire range of the measure.

**
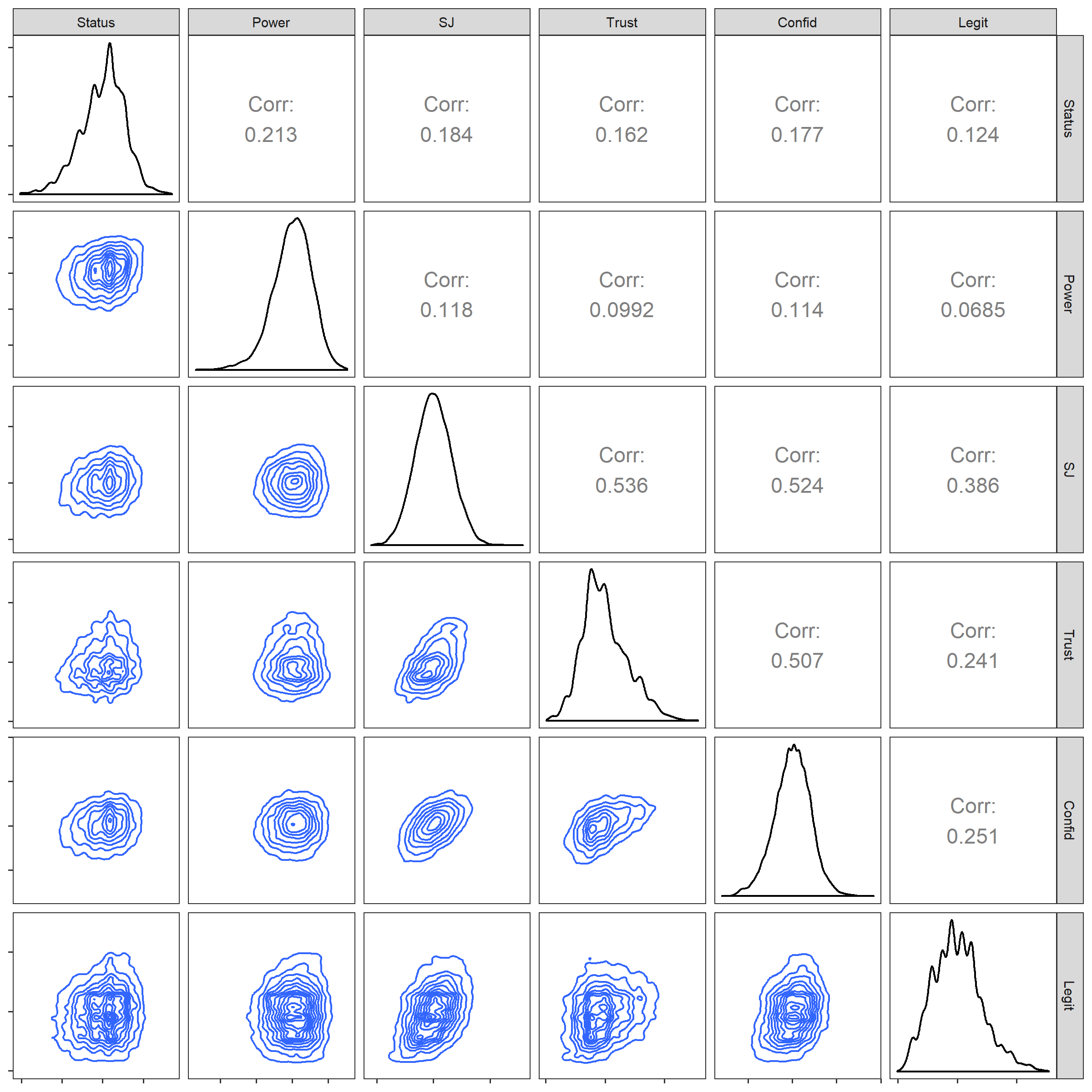
**

*Figure 1. Correlations (above the diagonal) and density plots (below the diagonal) between subjective status, sense of power, and the outcome variables. Density plots for each individual variable are on the diagonal. Density plots are a variant of a histogram. The area below the diagonal uses density plots between two variables. SJ = System Justification, Trust = Trust in Government, Confid = Confidence in Societal Institutions, Legit = Legitimacy of the status hierarchy.*

**
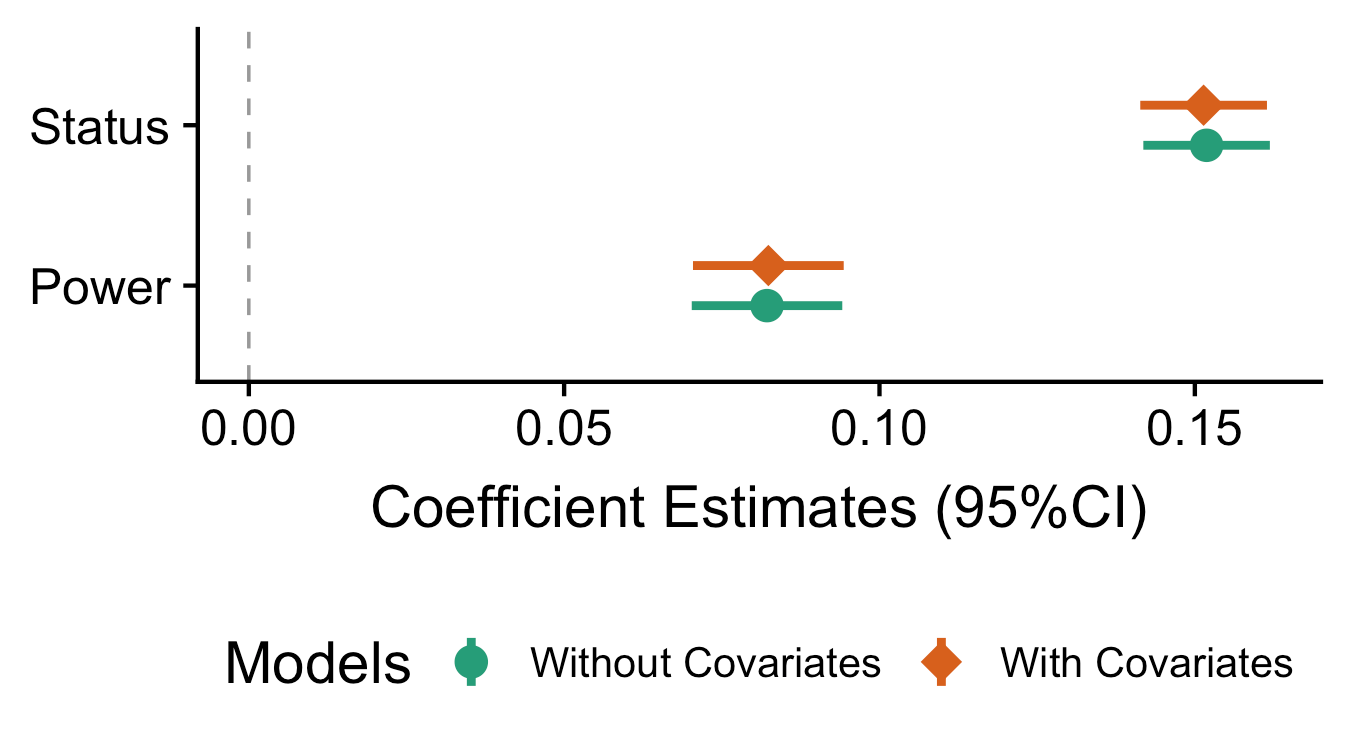
**

*Figure 2. Effects of subjective status and sense of power on perceived legitimacy. Error bars are 95% confidence intervals. A null effect is highlighted with the dashed horizontal line.*

Table 4. *Estimated slopes of subjective status and sense of power for each country from the multilevel model. Rows are sorted based on the size of the slope of subjective status on perceived legitimacy (the first column).*

|  | Perceived legitimacy | |
| --- | --- | --- |
| Country | Subjective Status | Sense of power |
| Netherlands | 0.27 | 0.18 |
| New Zealand | 0.21 | **-0.02** |
| Belgium | 0.20 | 0.03 |
| Ireland | 0.18 | 0.06 |
| Chile | 0.18 | **-0.02** |
| United Kingdom | 0.18 | 0.15 |
| United States | 0.17 | 0.13 |
| Spain | 0.16 | 0.08 |
| India | 0.15 | 0.03 |
| Greece | 0.15 | **-0.04** |
| Slovakia | 0.15 | 0.00 |
| Poland | 0.13 | 0.06 |
| Czech Republic | 0.13 | 0.07 |
| Australia | 0.12 | 0.11 |
| Turkey | 0.12 | 0.06 |
| France | 0.12 | 0.06 |
| Canada | 0.12 | 0.16 |
| Denmark | 0.11 | **-0.02** |
| Singapore | 0.10 | 0.12 |
| South Korea | 0.09 | 0.12 |
| Russia | 0.09 | 0.31 |
| Colombia | 0.08 | **-0.03** |
| Serbia | 0.08 | **-0.01** |
| Uruguay | 0.07 | 0.09 |
| Hungary | 0.07 | **-0.01** |
| Switzerland | 0.06 | **-0.07** |
| Italy | 0.04 | 0.04 |
| Lebanon | 0.04 | **-0.09** |
| Malaysia | 0.04 | 0.13 |
| Germany | 0.03 | **-0.04** |

*Note*: Bold values are values that are in the direction predicted by the status-legitimacy and power-legitimacy hypotheses. Slopes estimated using multilevel models described in the text.


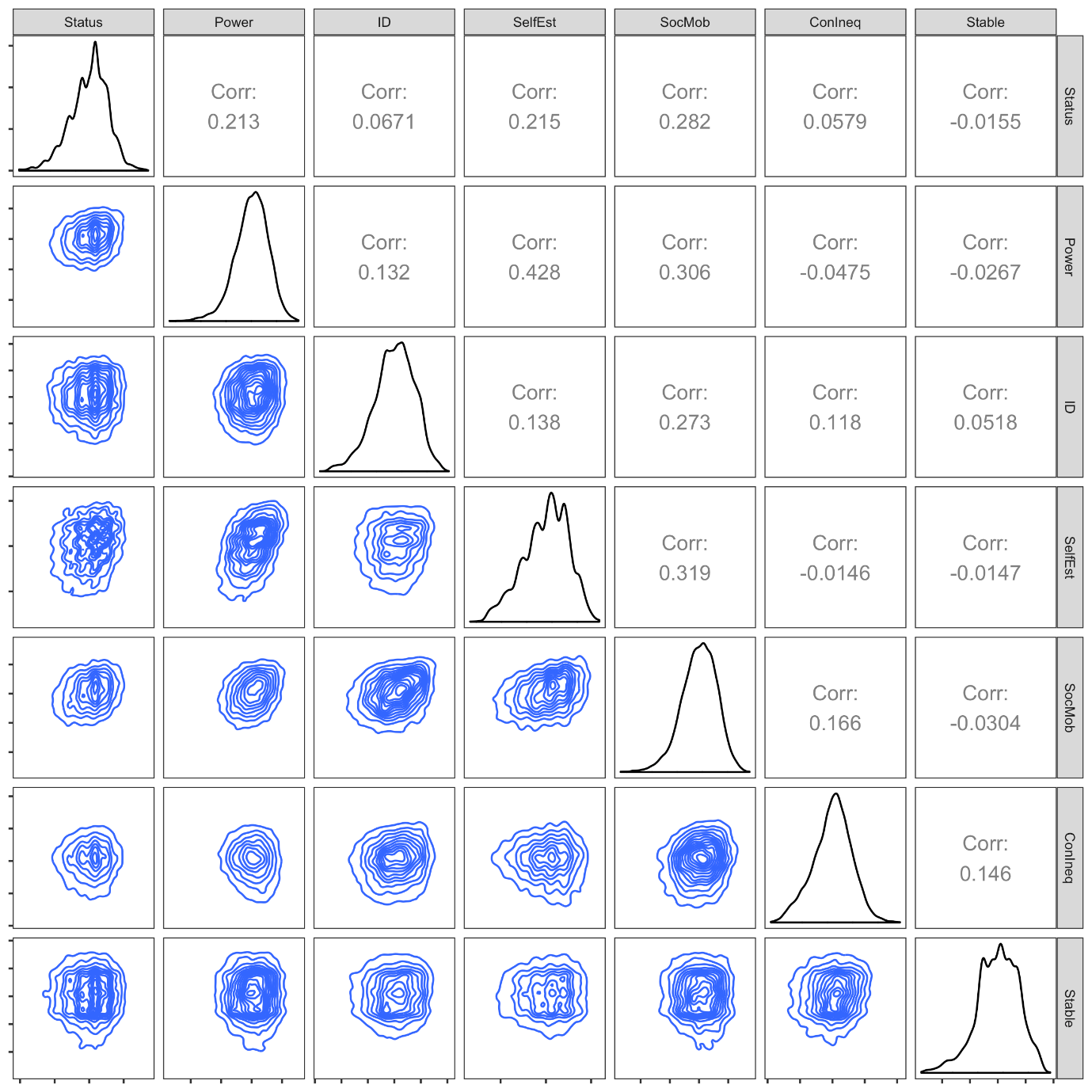


*Figure 3. Correlations (above the diagonal) and density plots (below the diagonal) between primary predictor variables and the individual level variables. All variables were country-mean centered before calculating the correlations. Density plots for each individual variable are on the diagonal. ID = Group identification, SelfEst = Self-esteem, SocMob = Social mobility, ConInqu = Contribution to inequality, Stable = Stability of the status hierarchy.*

**
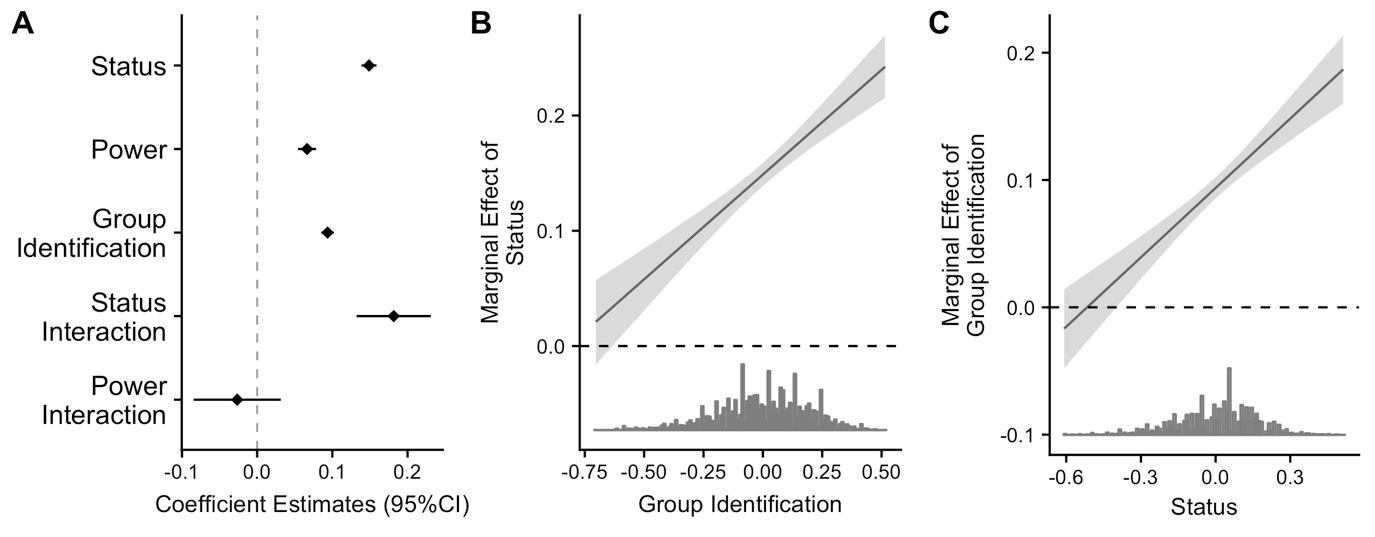
**

Figure 4. *Panel A: Effects of subjective status, sense of power, and group identification on perceived legitimacy. Covariates are included in the model. Error bars are 95% confidence intervals. Panel B: Marginal effect of subjective status on perceived legitimacy (y-axis) across the range of group identification (x-axis). Panel C: Marginal effect of group identification on perceived legitimacy (y-axis) across the range of subjective status (x-axis). For both Panels B and C, the grey band around the slope is the 95% confidence interval. In all panels a null effect is highlighted with the dashed line.*


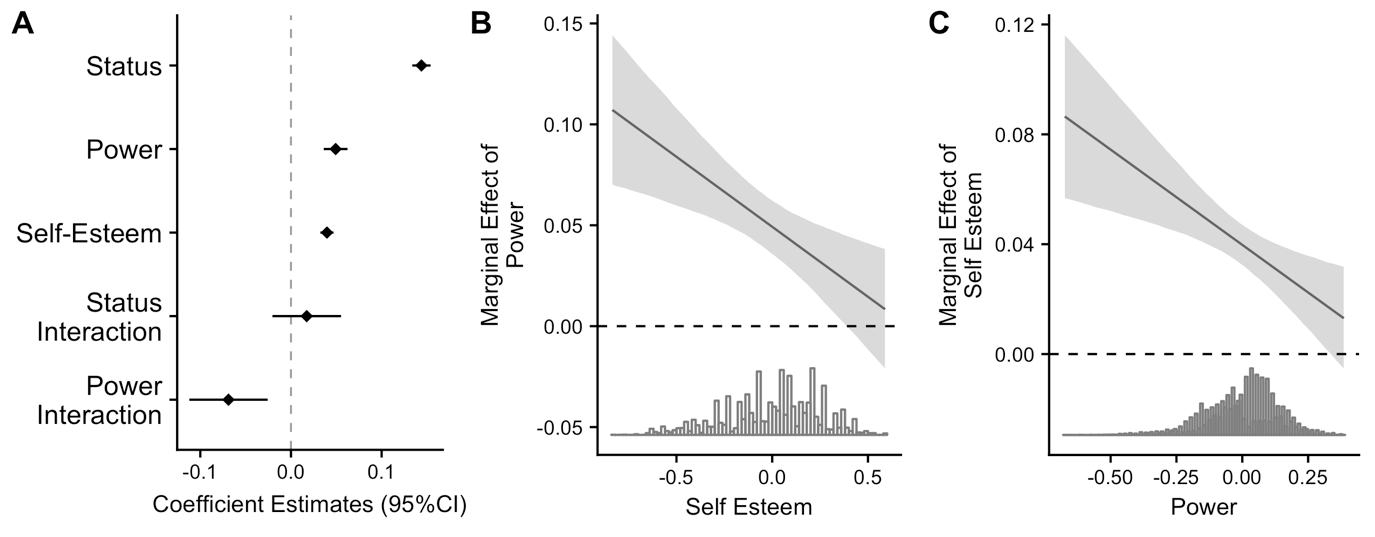


Figure 5. *Panel A: Effects of subjective status, sense of power, and self-esteem on perceived legitimacy. Covariates are included in the model. Error bars are 95% confidence intervals. Panel B: Marginal effect of sense of power on perceived legitimacy (y-axis) across the range of self-esteem (x-axis). Panel C: Marginal effect of self-esteem on perceived legitimacy (y-axis) across the range of sense of power (x-axis). For both Panels B and C, the grey band around the slope is the 95% confidence interval. In all panels a null effect is highlighted with the dashed line.*


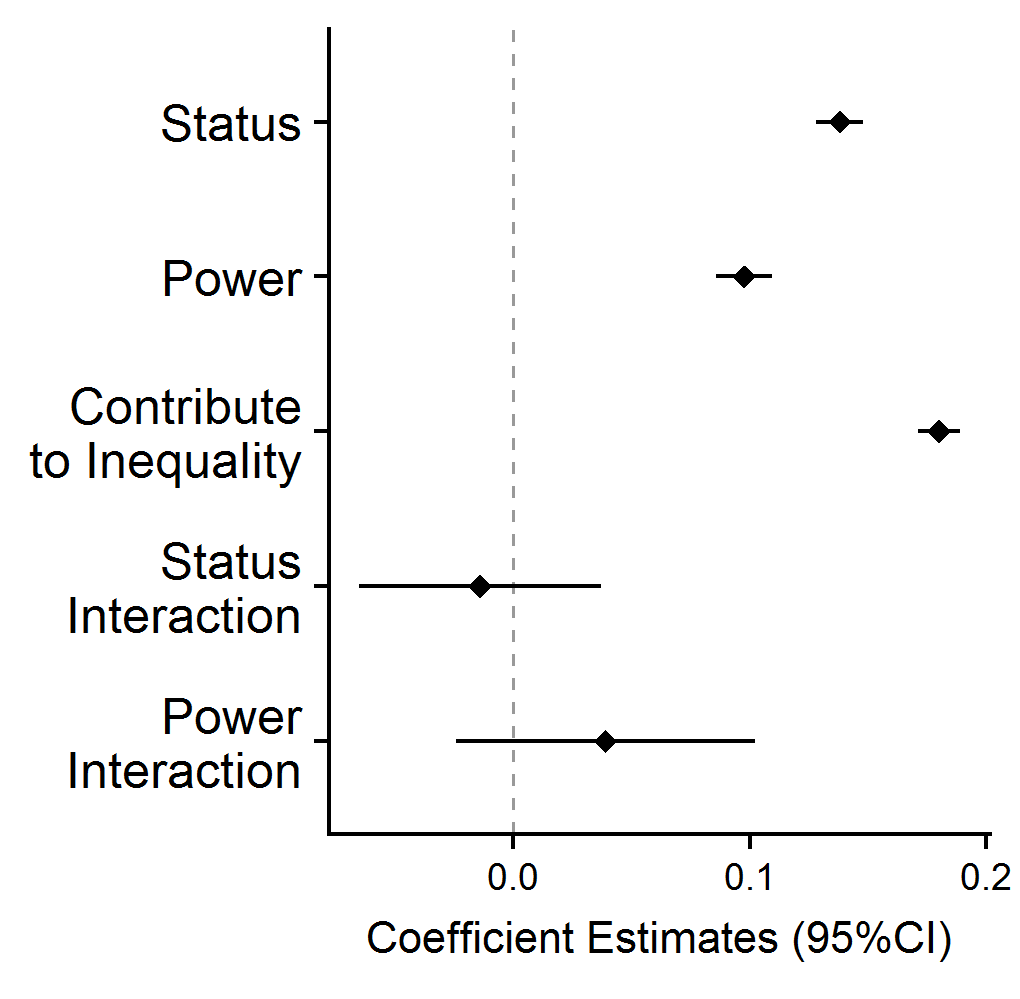


Figure 6. *Effects of subjective status, sense of power, and contributing to inequality on perceived legitimacy. Covariates are included in the model. Error bars are 95% confidence intervals.*


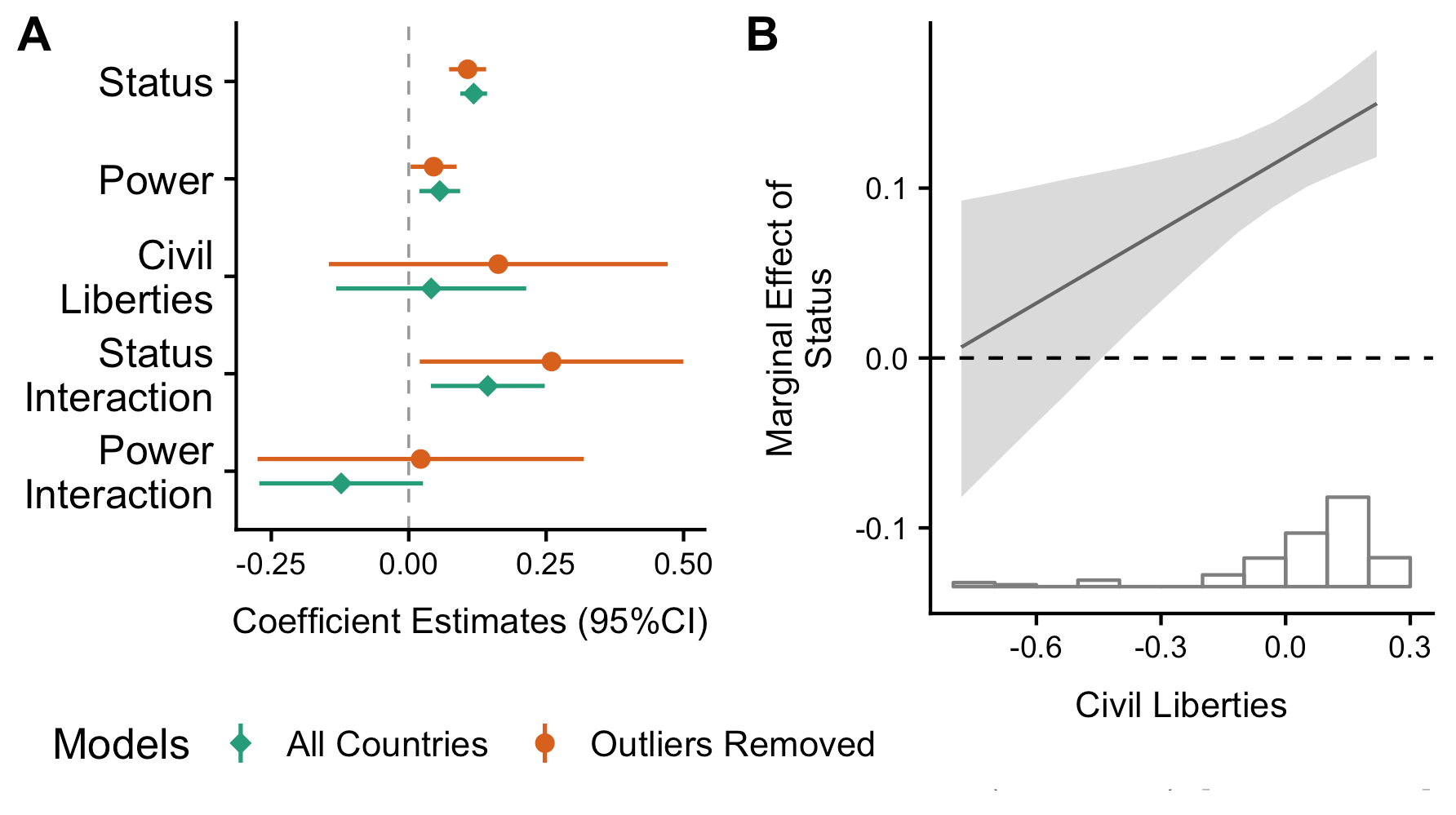


Figure 7. *Panel A: Effects of subjective status, sense of power, and civil liberties on perceived legitimacy. Covariates are included in the model. Error bars are 95% confidence intervals. Panel B: Marginal effect of subjective status on perceived legitimacy (y-axis) across the range of civil liberties (x-axis). The grey band around the slope is the 95% confidence interval. In all panels a null effect is highlighted with the dashed line. See footnote 10 for outlier explanation.*


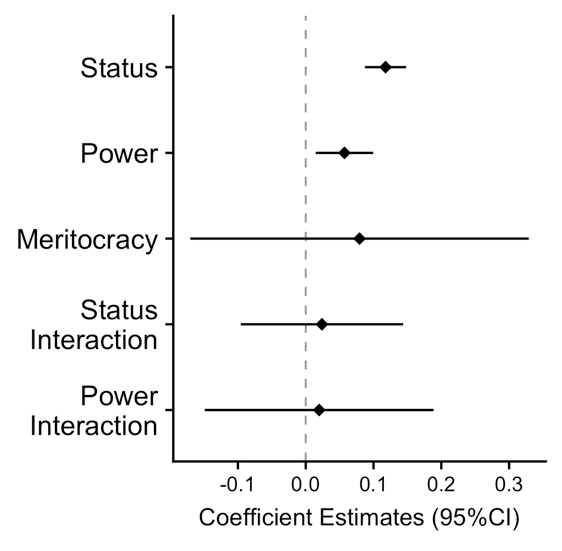


Figure 8. *Effects of subjective status, sense of power, and meritocracy on perceived legitimacy. Covariates are included in the model. Error bars are 95% confidence intervals.*


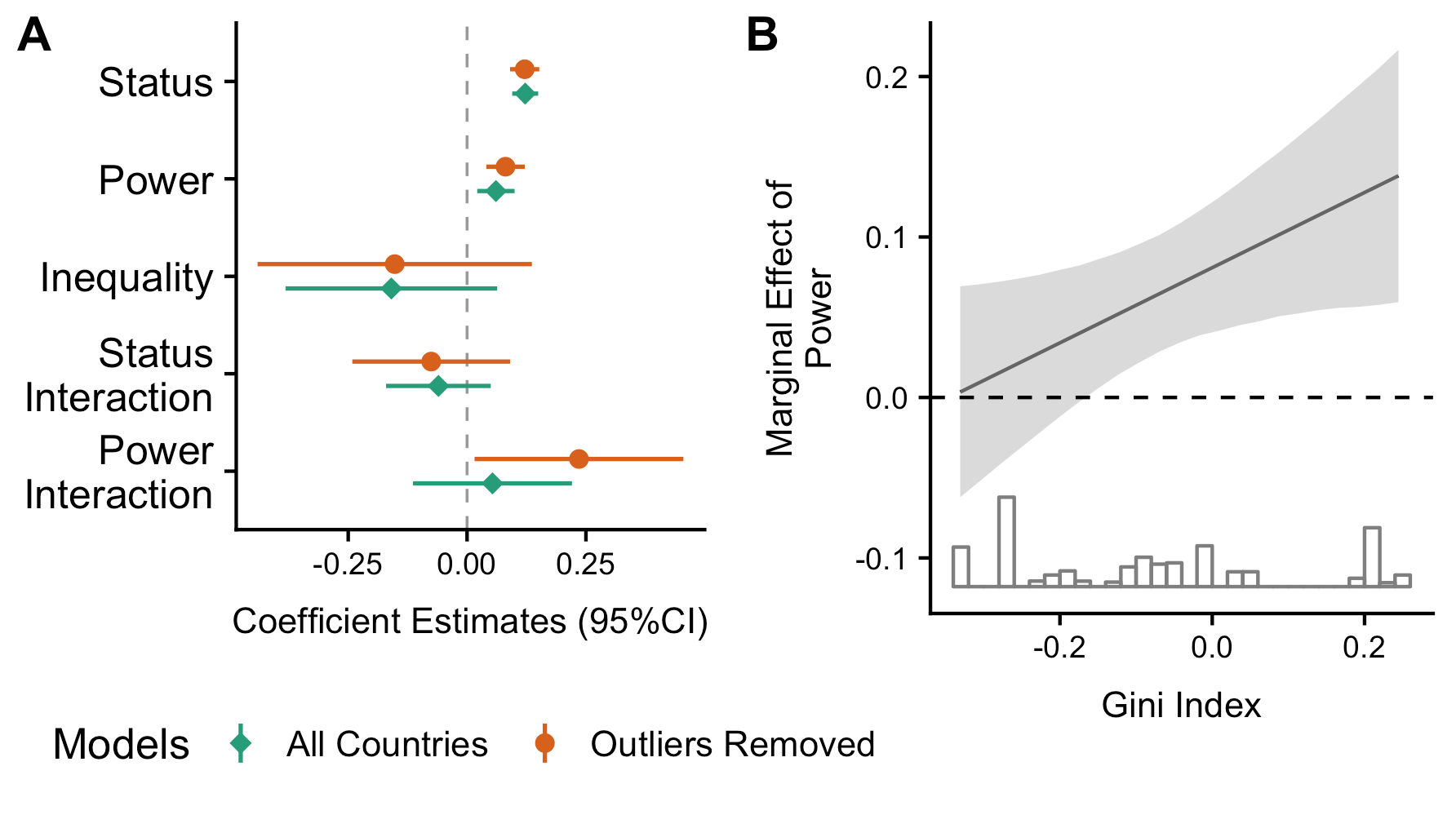


Figure 9. *Panel A: Effects of subjective status, sense of power, and inequality on perceived legitimacy. Covariates are included in the model. Error bars are 95% confidence intervals. Panel B: Marginal effect of sense of power on perceived legitimacy (y-axis) across the range of inequality when outliers are removed (x-axis). The grey band around the slope is the 95% confidence interval. In all panels a null effect is highlighted with the dashed line. See footnote 10 for outlier explanation.*


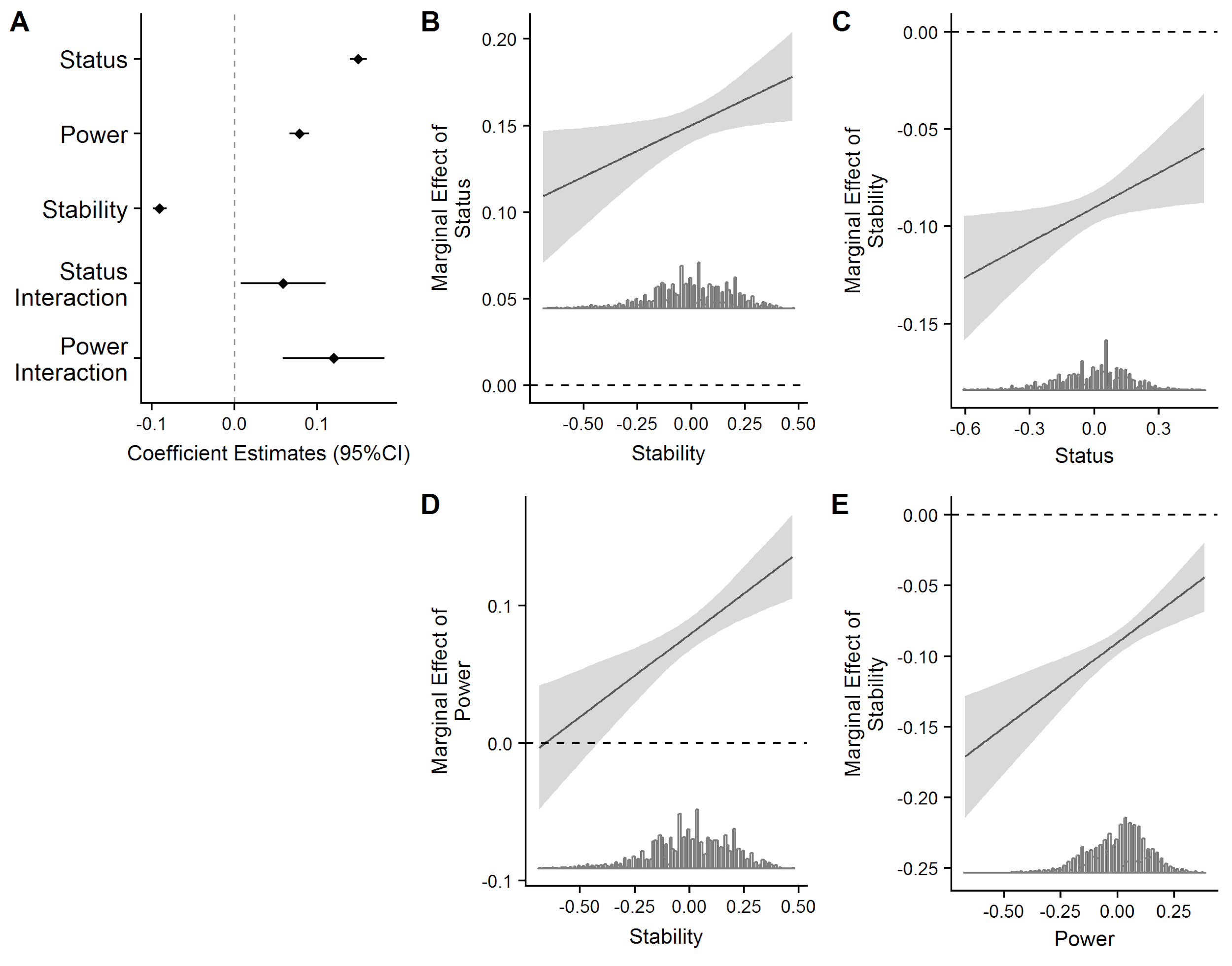


Figure 10. *Panel A: Effects of subjective status, sense of power, and stability of the subjective status hierarchy on perceived legitimacy. Covariates are included in the model. Error bars are 95% confidence intervals. Panel B: Marginal effect of subjective status on perceived legitimacy (y-axis) across the range of stability (x-axis). Panel C: Marginal effect of stability on perceived legitimacy (y-axis) across the range of subjective status (x-axis). Panel D: Marginal effect of sense of power on perceived legitimacy (y-axis) across the range of stability (x-axis). Panel E: Marginal effect of stability on perceived legitimacy (y-axis) across the range of sense of power (x-axis). For Panels B, C, and D the grey band around the slope is the 95% confidence interval. In all panels a null effect is highlighted with the dashed line.*


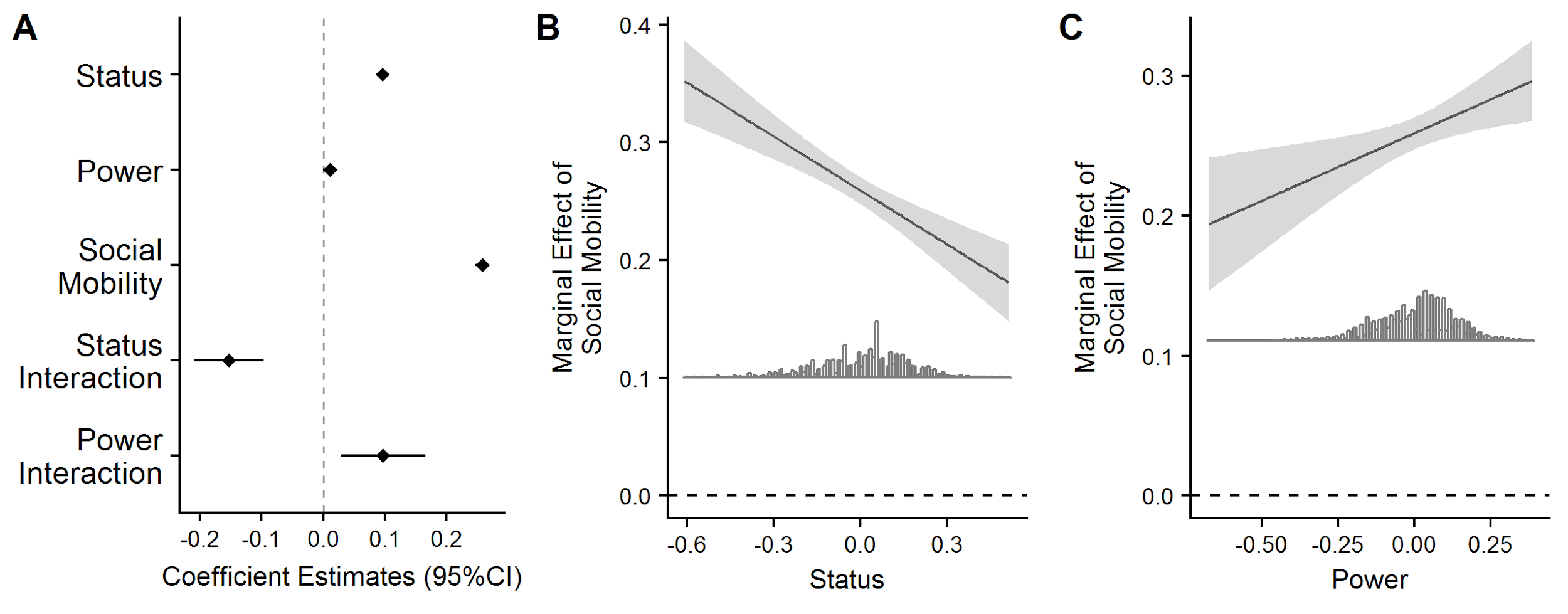


Figure 11*. Panel A: Effects of subjective status, sense of power, and social mobility on perceived legitimacy. Covariates are included in the model. Error bars are 95% confidence intervals. Panel B: Marginal effect of social mobility on perceived legitimacy (y-axis) across the range of subjective status (x-axis). Panel C: Marginal effect of social mobility on perceived legitimacy (y-axis) across the range of sense of power (x-axis). For both Panels B and C, the grey band around the slope is the 95% confidence interval. In all panels a null effect is highlighted with the dashed line.*

Table 5. *Summary of moderation hypotheses (approach #1) and whether they were supported when subjective status or power was the predictor.*

|  | **Predictor** | |  |
| --- | --- | --- | --- |
| **Moderator Hypothesis** | ***Status*** | ***Power*** |  |
| ***Reduce Threat*** |  |  |  |
| Identification-Moderation Hypothesis | ✓ | ✗ |  |
| Self-Esteem-Moderation Hypothesis | ✗ | ✗^op^ |  |
| ***Increase Threat Through Dissonance*** |  |  |  |
| Inequality Contribution-Moderation Hypothesis | ✗ | ✗ |  |
| Civil Liberties Hypothesis | ✗^op^ | ✗ |  |
| Meritocracy Hypothesis | ✗ | ✗ |  |
| SJT Inequality Hypothesis | ✗ | ✗ |  |
| ***Structural Factors that Affect Threat*** |  |  |  |
| SIT Inequality Hypothesis | ✗ | ✓ ^o^ |  |
| Stability-Moderator Hypothesis | ✗^op^ | ✗^op^ |  |
| *Note*: ✓ = indicates support and partial support for the hypothesis, ✗ = indicates no support for the hypothesis. . ✓ ^o^ = indicates a significant interaction effect in the predicted direction, but only after removing outliers. ✗^op^ = indicates a significant interaction effect in the opposite direction. SJT = System justification theory, SIT = Social identity theory. | | | |

Table 6. *Summary of predictor hypotheses (approach #2) and whether they were supported for people low in subjective status or in sense of power.*

|  | **For people low in…** | |  |
| --- | --- | --- | --- |
| **Predictor Hypothesis** | ***Status*** | ***Power*** |  |
| Social Mobility-Legitimacy Hypothesis | ✓ | ✓ |  |
| Stability-Legitimacy Hypothesis | ✗^op^ | ✗^op^ |  |
| Identification-Legitimacy Hypothesis | ✗ | ✗^op^ |  |
| Self-Esteem Legitimacy Hypothesis | ✗^op^ | ✗^op^ |  |
| *Note*: ✓ = indicates support and partial support for the hypothesis, ✗ = indicates no support for the hypothesis. ✗^op^ = indications a significant interaction effect in the opposite direction. SJT = System justification theory, SIT = Social identity theory | | | |

# Analyses for Admiration and Contempt for High Status People

We included measures of admiration and contempt for high status and low status people. Bias for high status groups relatively to low status groups has been a marker of system justification processes. These measures may capture less direct forms of perceived legitimacy.

To measure participants’ admiration and contempt for high status people, we first had people read a short paragraph of text describing the social status ladder (the same ladder used to measure status). Then, to measure admiration, we asked “How much admiration do you feel towards people at the very top of the ladder?” To measure contempt, we asked “How much contempt do you feel towards people at the very top of the ladder?” For analyses of these two items, we controlled for admiration or contempt of low status people that was measured using identical items, but referred to people “at the very bottom of the ladder.”

## Main effects

Because contempt and admiration were only weakly correlated, we treated these as separate three-level multilevel models: persons nested in samples/labs, nested in countries. In these models we included as a covariate people’s admiration and contempt for low status people in their respective models (admiration for low status people in the admiration model, contempt for low status people in the contempt model; country-mean centered).

The results of the models testing the main effects are in Figure S2. The effects largely mirror those of perceived legitimacy. People with higher status and sense of power have more admiration and less contempt for high status people. This is inconsistent with the Status Legitimacy and the Power Legitimacy Hypotheses. The estimated slopes within each country are in Table S1. They are calculated in the same manner as in the main text.


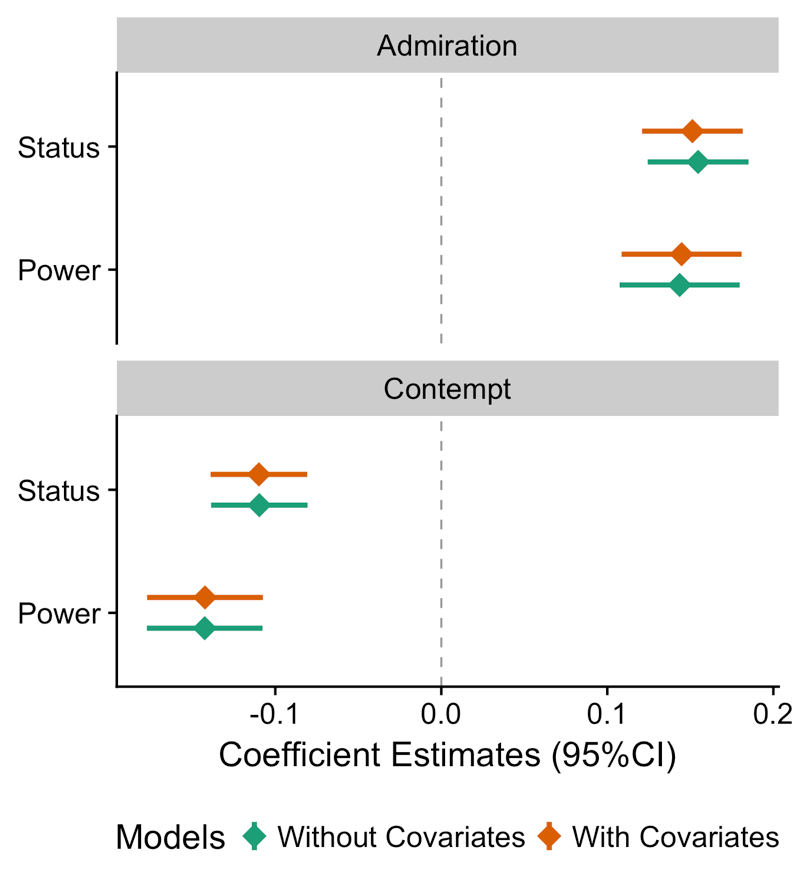


*Figure S2. Effects of status and sense of power on admiration and contempt for high status people (controlling for admiration and contempt for low status people, respectively). Error bars are 95% confidence intervals. A null effect is highlighted with the dashed horizontal line.*

*Table 2. Estimated slopes of status and power for each country for each of the three outcome variables. Rows are sorted based on the size of the slope of status on legitimacy (the first column).*

|  | Admiration | |  | Contempt | |
| --- | --- | --- | --- | --- | --- |
| Country | Status | Power |  | Status | Power |
| Netherlands | 0.17 | 0.18 |  | -0.20 | -0.17 |
| New Zealand | 0.08 | 0.06 |  | -0.02 | -0.10 |
| Belgium | 0.13 | 0.12 |  | -0.22 | -0.20 |
| Ireland | 0.08 | 0.05 |  | -0.09 | -0.15 |
| Chile | 0.15 | 0.14 |  | -0.14 | -0.18 |
| United Kingdom | 0.13 | 0.13 |  | -0.05 | -0.13 |
| United States | 0.20 | 0.22 |  | -0.03 | -0.11 |
| Spain | 0.20 | 0.21 |  | -0.26 | -0.16 |
| India | 0.16 | 0.16 |  | **0.12** | -0.09 |
| Greece | 0.23 | 0.25 |  | -0.19 | -0.09 |
| Slovakia | 0.13 | 0.13 |  | -0.15 | -0.19 |
| Poland | 0.10 | 0.08 |  | -0.07 | -0.06 |
| Czech Republic | 0.09 | 0.06 |  | -0.21 | -0.20 |
| Australia | 0.18 | 0.18 |  | -0.06 | -0.11 |
| Turkey | 0.22 | 0.24 |  | -0.09 | -0.15 |
| France | 0.12 | 0.11 |  | -0.16 | -0.21 |
| Canada | 0.13 | 0.12 |  | -0.09 | -0.10 |
| Denmark | 0.12 | 0.10 |  | -0.13 | -0.13 |
| Singapore | 0.11 | 0.10 |  | -0.04 | -0.12 |
| South Korea | 0.08 | 0.06 |  | -0.20 | -0.13 |
| Russia | 0.18 | 0.19 |  | -0.15 | -0.17 |
| Colombia | 0.11 | 0.09 |  | -0.07 | -0.12 |
| Serbia | 0.14 | 0.14 |  | -0.16 | -0.14 |
| Uruguay | 0.15 | 0.15 |  | -0.14 | -0.14 |
| Hungary | 0.18 | 0.19 |  | -0.04 | -0.13 |
| Switzerland | 0.16 | 0.16 |  | -0.20 | -0.16 |
| Italy | 0.16 | 0.16 |  | -0.04 | -0.13 |
| Lebanon | 0.11 | 0.10 |  | -0.04 | -0.15 |
| Malaysia | 0.13 | 0.12 |  | -0.03 | -0.12 |
| Germany | 0.06 | 0.03 |  | -0.09 | -0.14 |

*Note*: Bold values the values that are in the direction predicted by the status-legitimacy and power-legitimacy hypotheses. Slopes estimated using multilevel models described in the text.

## Assessing Moderators and Predictors

We tested for moderation effects by each of our individual-level and societal-level moderators by building on the model including covariates. For each moderator, we conducted a separate model in which we added the moderator and its interaction between status and power. The results of the models are in Figures S3 – S6.

The significant interactions were probed to see the effect of status and sense of power at different levels of the moderators, as well as to see the effect of the moderator at low levels of status and sense of power. This allows us to test the moderators as both predictors and boundary conditions (see main text for justification and description of approach). The moderators are probed in Figures S7 – S13. The results are summarized in Table S2 and Table S3.

Table S2. *Summary of moderation hypotheses (approach #1) and whether they were supported when status or power was the predictor.*

|  | **Outcome = Admiration**  **Predictor** | | **Outcome = Contempt**  **Predictor** | |  |
| --- | --- | --- | --- | --- | --- |
| **Moderator Hypothesis** | ***Status*** | ***Power*** | ***Status*** | ***Power*** |  |
| ***Reduce Threat*** |  |  |  |  |  |
| Identification-Moderation Hypothesis | ✓^+^ | ✗ | ✓ | ✗ |  |
| Salience-Moderation Hypothesis | ✗ | ✗ | ✗ | ✗ |  |
| Self-Esteem-Moderation Hypothesis | ✓ | ✗ | ✗ | ✗ |  |
| ***Increase Threat Through Dissonance*** |  |  |  |  |  |
| Inequality Contribution-Moderation Hypothesis | ✗ | ✓ | ✗ | ✗ ^o^ |  |
| Civil Liberties Hypothesis | ✗ | ✓ | ✗ | ✗ |  |
| Meritocracy Hypothesis | ✗ | ✗ | ✗ ^o^ | ✗ ^o^ |  |
| SJT Inequality Hypothesis | ✗ | ✗ ^o^ | ✗ | ✗ |  |
| ***Structural Factors that Affect Threat*** |  |  |  |  |  |
| SIT Inequality Hypothesis | ✗ | ✓ | ✗ | ✗ |  |
| Stability-Moderator Hypothesis | ✗ ^o^ | ✗ | ✗ | ✗ |  |
| *Note*: ✓ = indicates support and partial support for the hypothesis, ✗ = indicates no support for the hypothesis. ✓ ^+^ = indicates a significant interaction effect in the predicted direction, and evidence for the status-legitimacy hypothesis where expected. ✗^o^ = indicates a significant interaction effect in the opposite direction. SJT = System justification theory, SIT = Social identity theory | | | | | |

Table S3

*Summary of predictor hypotheses and whether they were supported for people low in status or in sense of power and admiration or contempt was the outcome variable.*

|  | **Outcome = Admiration**  **For people low in…** | | **Outcome = Contempt**  **For people low in…** | |  |
| --- | --- | --- | --- | --- | --- |
| **Predictor Hypothesis** | ***Status*** | ***Power*** | ***Status*** | ***Power*** |  |
| Social Mobility-Legitimacy Hypothesis | ✓ | ✓ | ✓ | ✗ |  |
| Stability-Legitimacy Hypothesis | ✗ | ✓ | ✗ ^o^ | ✗ ^o^ |  |
| Identification-Legitimacy Hypothesis | ✗ | ✗ ^o^ | ✓ | ✓ |  |
| Salience-Predictor Hypothesis | ✗ ^o^ | ✗ ^o^ | ✗ ^o^ | ✗ ^o^ |  |
| Self-Esteem Legitimacy Hypothesis | ✗ | ✗ ^o^ | ✗ ^o^ | ✗ ^o^ |  |
| *Note*: ✓ = indicates support and partial support for the hypothesis, ✗ = indicates no support for the hypothesis. ✓ ^+^ = indicates a significant interaction effect in the predicted direction, and evidence for the status-legitimacy hypothesis where expected. ✗^o^ = indicates a significant interaction effect in the opposite direction. SJT = System justification theory, SIT = Social identity theory | | | | | |


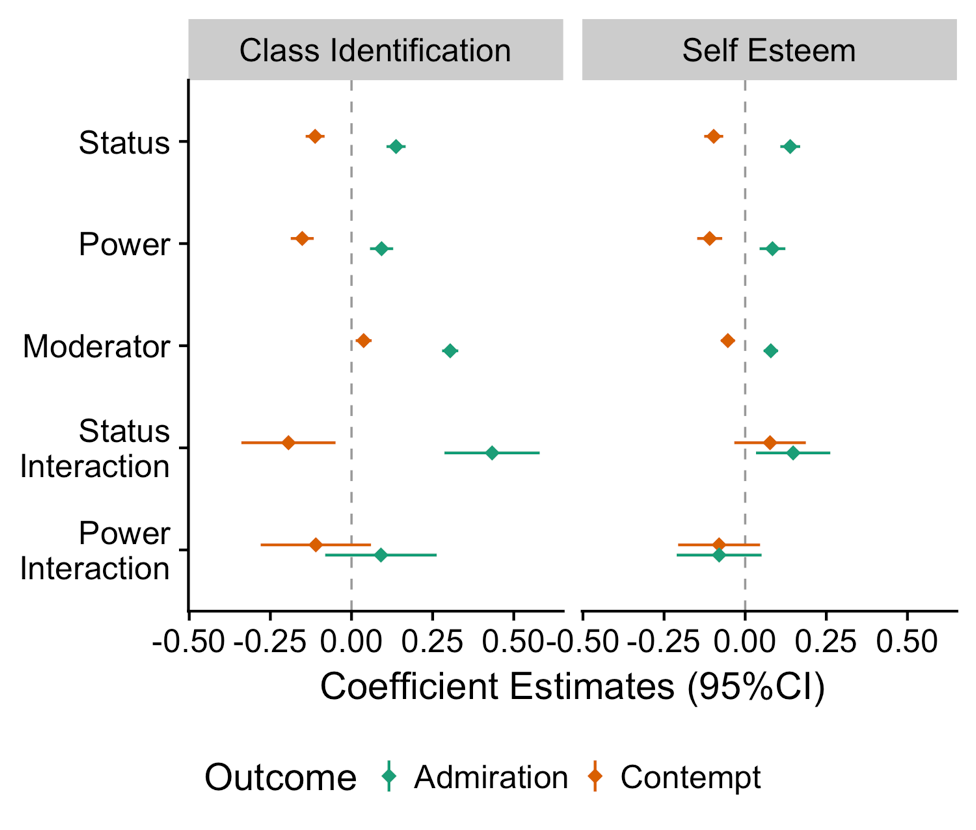


*Figure S3. Effects of status, sense of power, class identification, and self-esteem moderators on admiration and contempt. All models include covariates.*


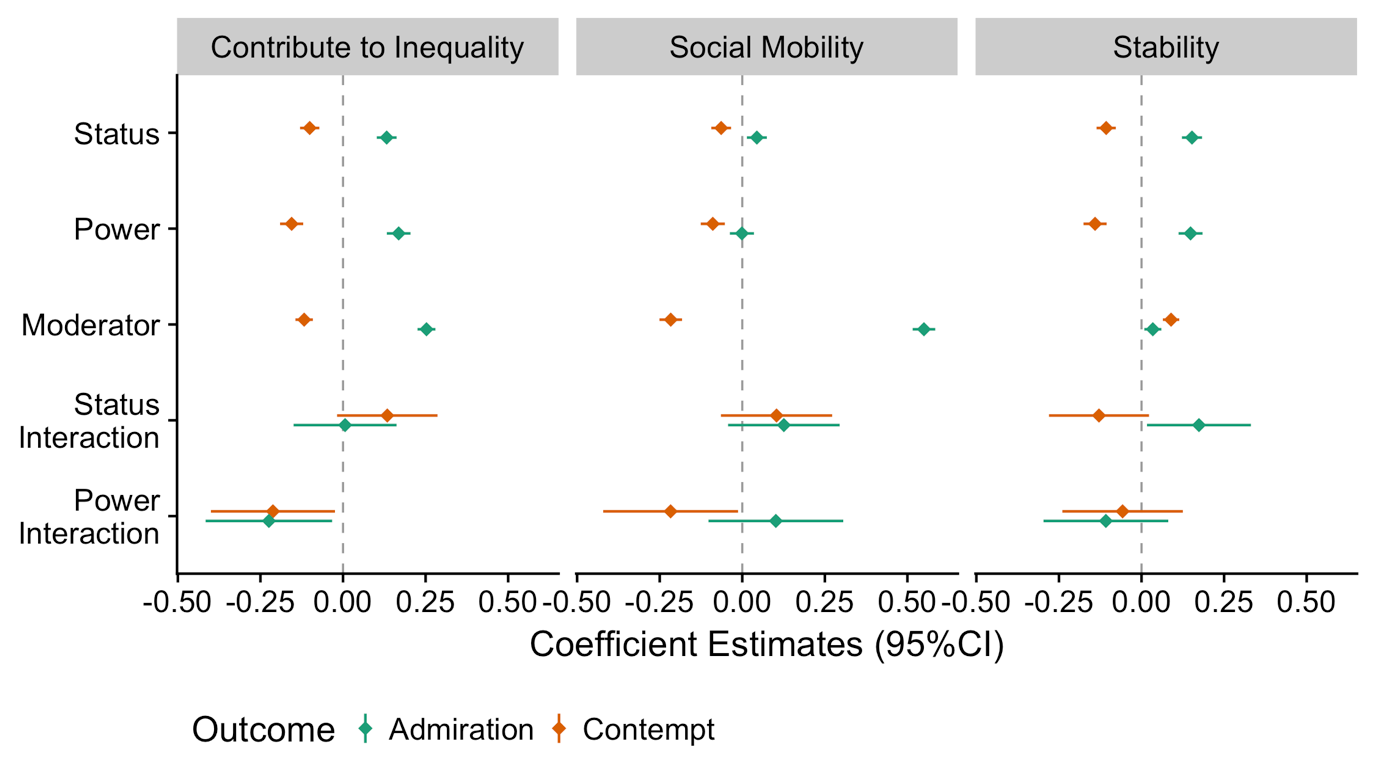


*Figure S4. Effects of status, sense of power, contribute to inequality, social mobility, and stability moderators on admiration and contempt. All models include covariates.*


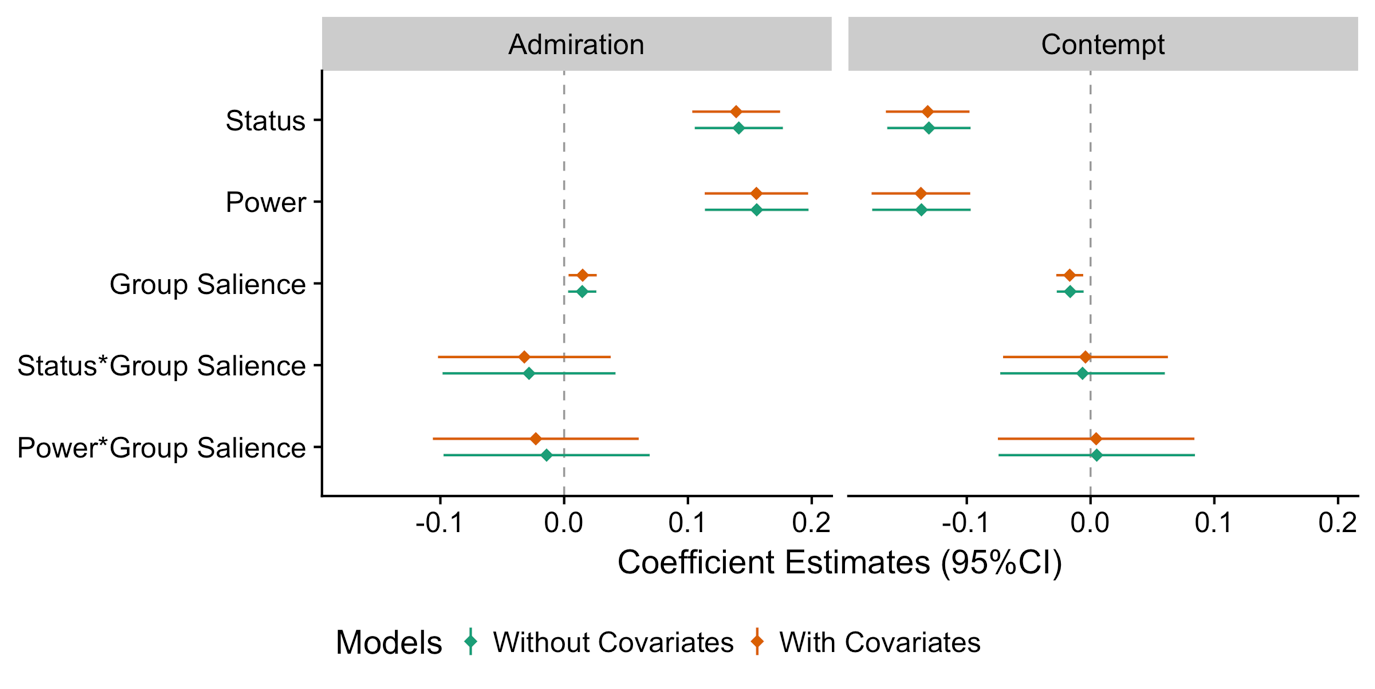


*Figure S5. Effects of status, sense of power, and experimentally manipulated group salience on admiration and contempt.*


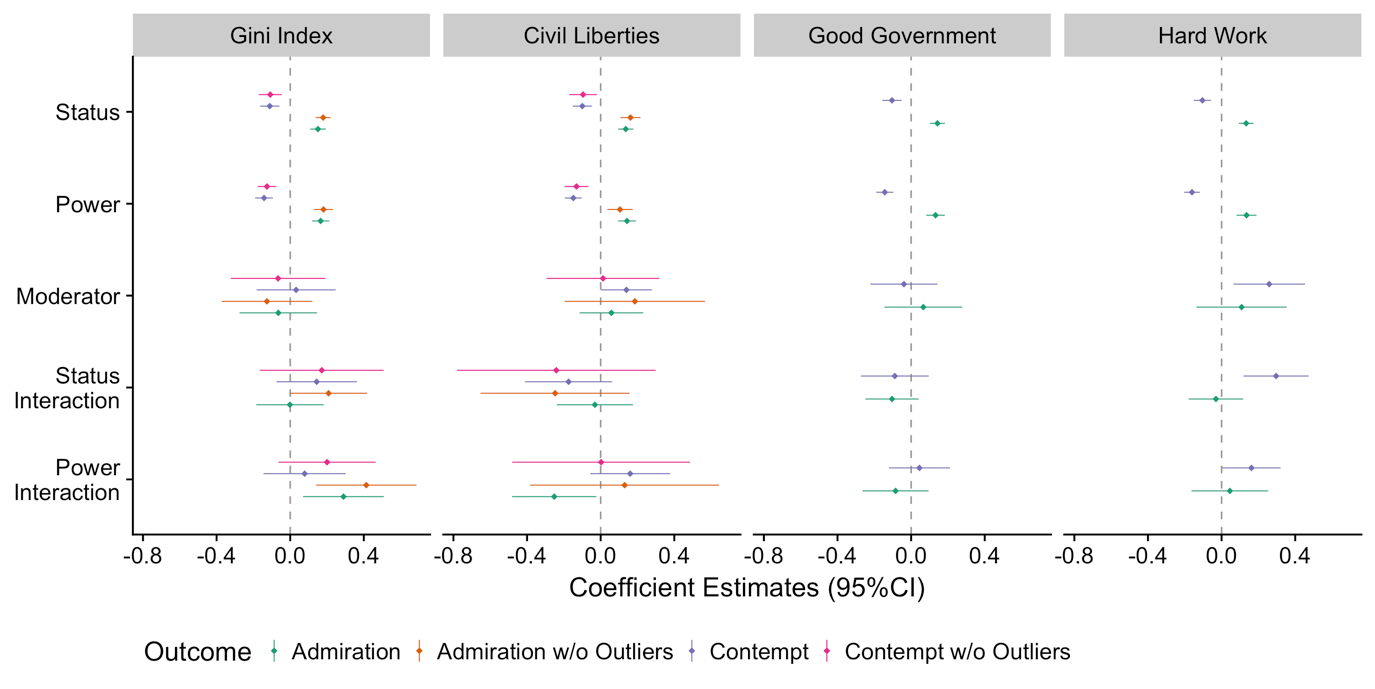


*Figure S6. Effects of status, sense of power, and the four country-level moderators on admiration and contempt. All models include covariates.*


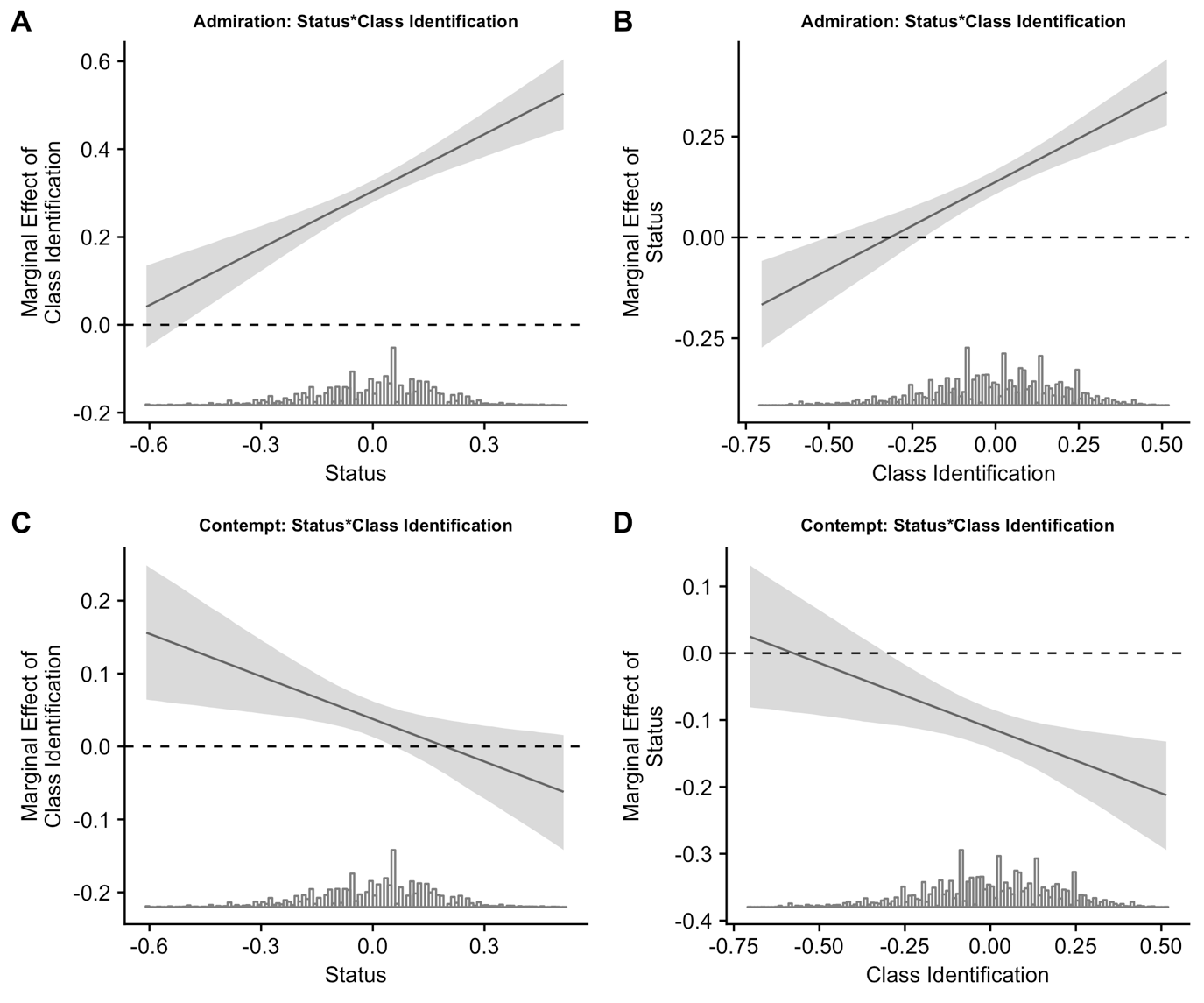


*Figure S7. Marginal effect plots of status and class identification on admiration and contempt. X-axis is the moderator variable with a histogram of the variables’ distribution. The y-axis is the effect of the predictor variable on admiration or contempt at different levels of the moderator variable. The grey band around the slope is the 95% confidence interval. A null effect is highlighted with the dashed horizontal line.*


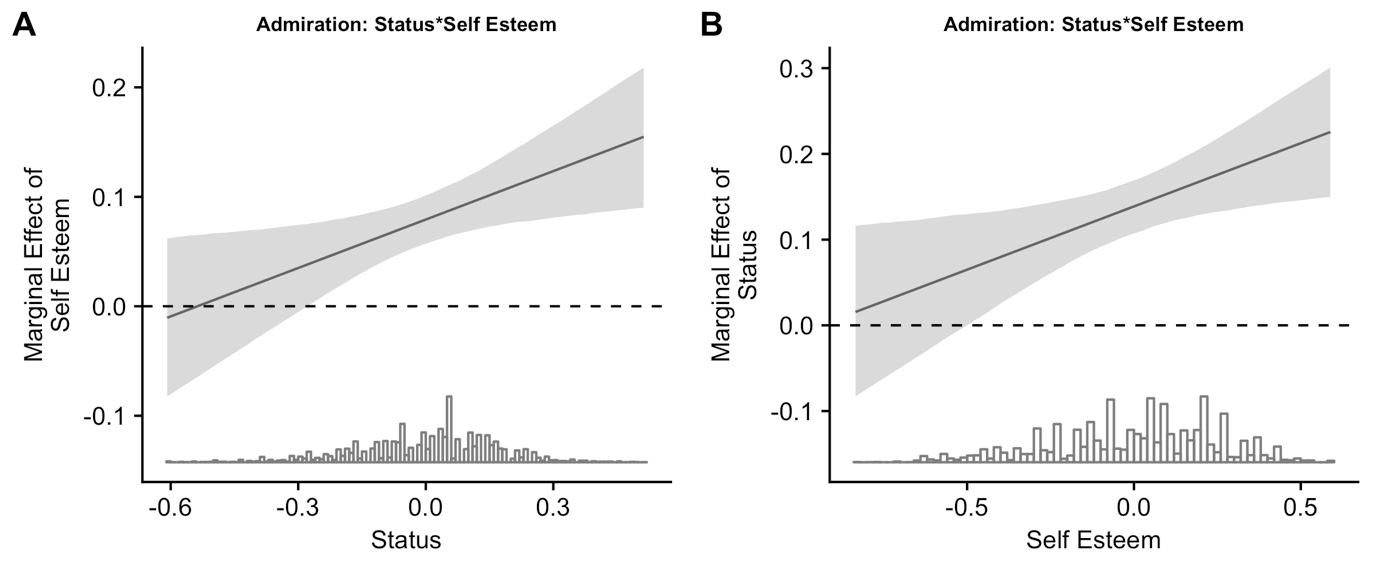


*Figure S8. Marginal effect plots of status and self-esteem on admiration. X-axis is the moderator variable with a histogram of the variables’ distribution. The y-axis is the effect of the predictor variable on admiration at different levels of the moderator variable. The grey band around the slope is the 95% confidence interval. A null effect is highlighted with the dashed horizontal line.*


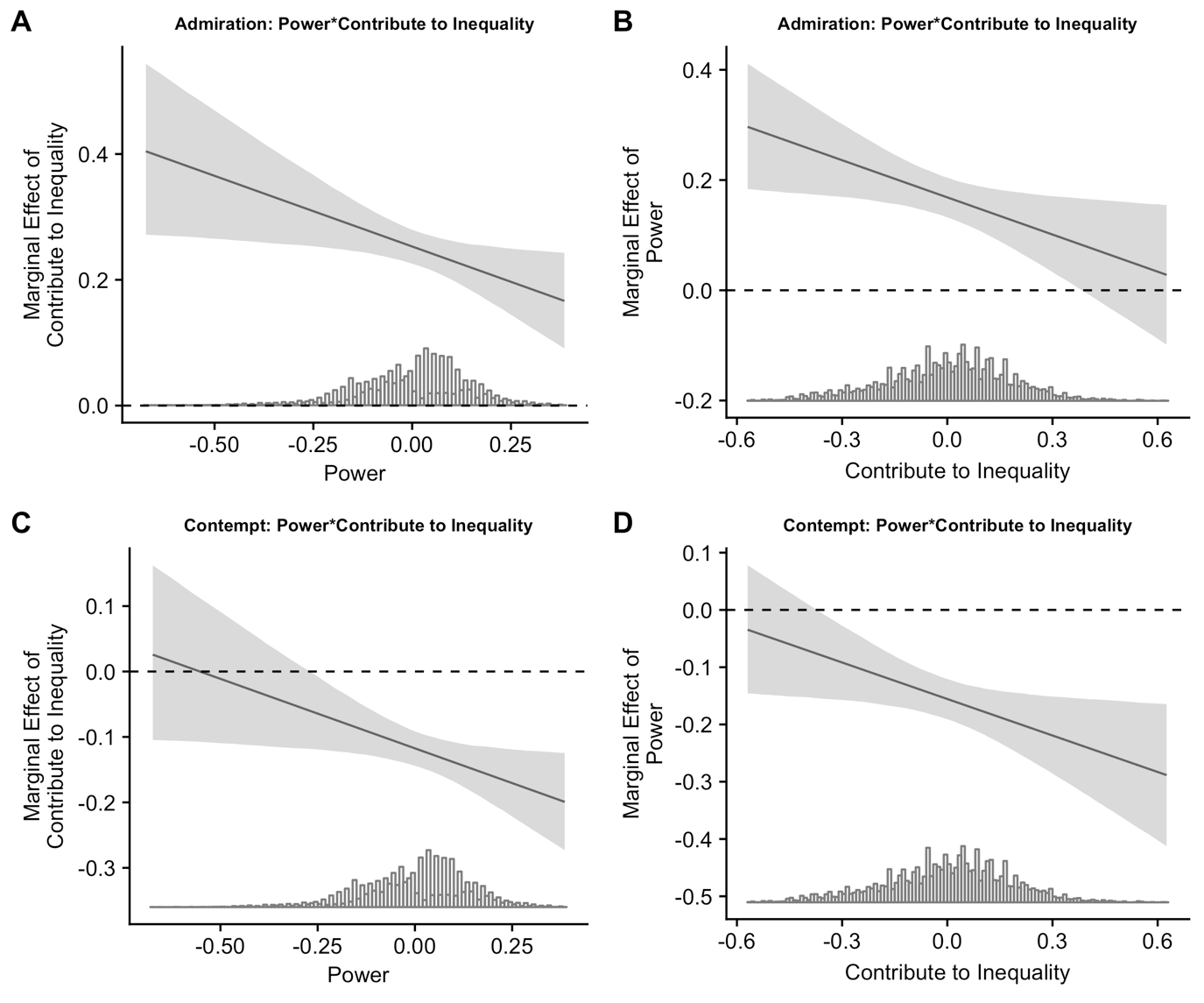


*Figure S9. Marginal effect plots of power and contribution to inequality on admiration and contempt. X-axis is the moderator variable with a histogram of the variables’ distribution. The y-axis is the effect of the predictor variable on admiration and contempt at different levels of the moderator variable. The grey band around the slope is the 95% confidence interval. A null effect is highlighted with the dashed horizontal line.*


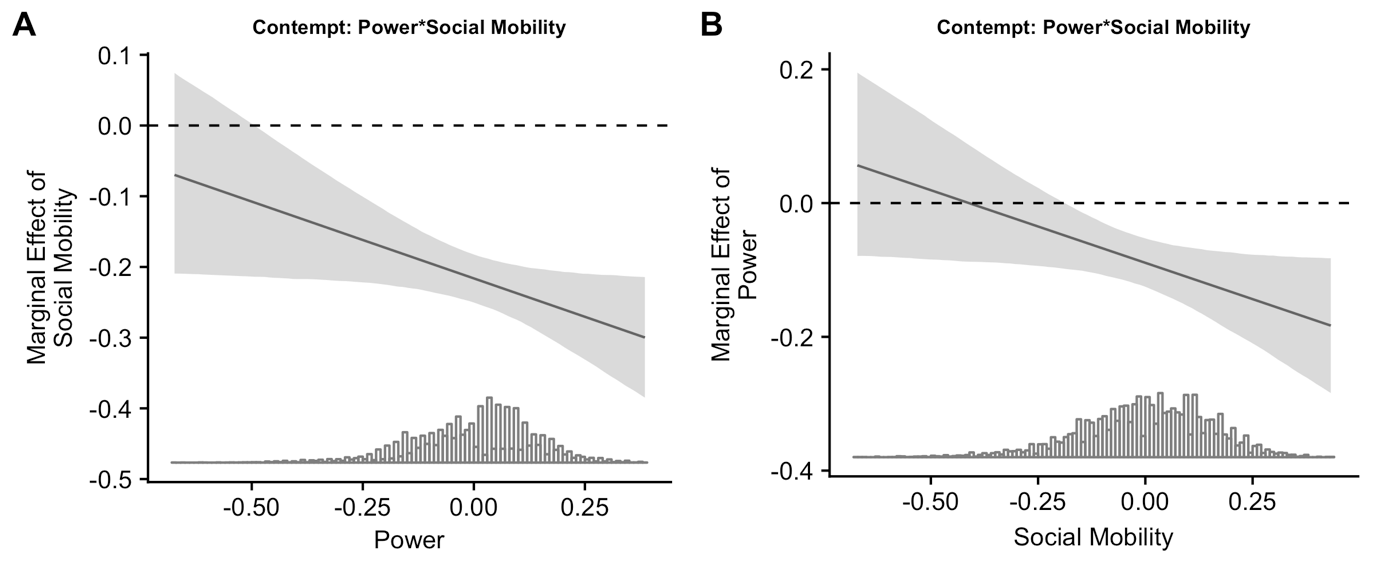


*Figure S10. Marginal effect plots of power and social mobility on contempt. X-axis is the moderator variable with a histogram of the variables’ distribution. The y-axis is the effect of the predictor variable on contempt at different levels of the moderator variable. The grey band around the slope is the 95% confidence interval. A null effect is highlighted with the dashed horizontal line.*


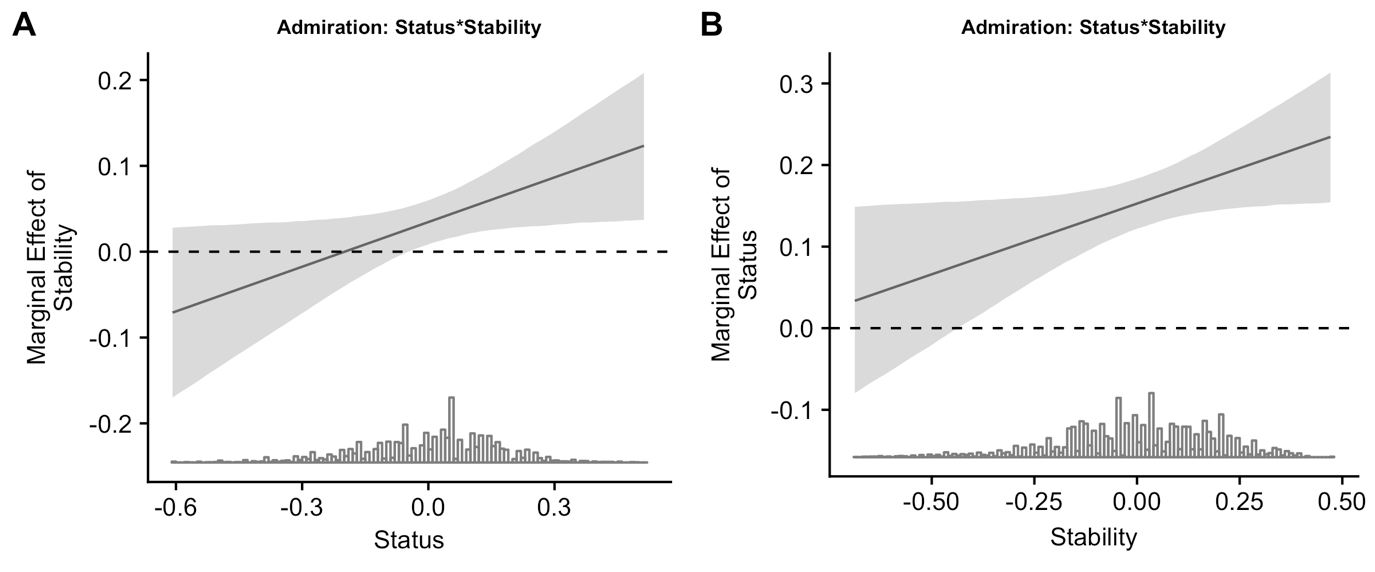


*Figure S11. Marginal effect plots of status and stability on admiration. X-axis is the moderator variable with a histogram of the variables’ distribution. The y-axis is the effect of the predictor variable on admiration at different levels of the moderator variable. The grey band around the slope is the 95% confidence interval. A null effect is highlighted with the dashed horizontal line.*


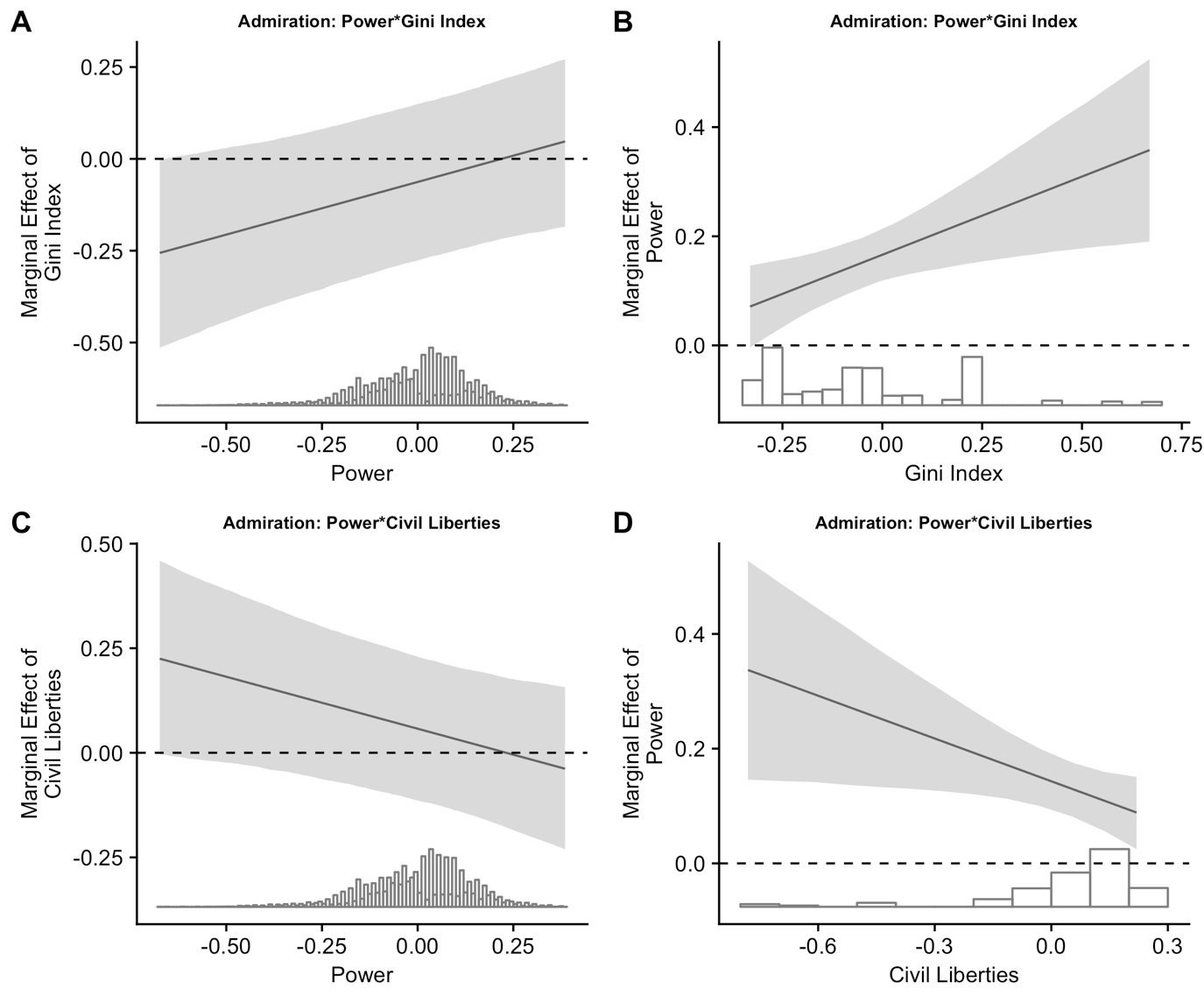


*Figure S12. Marginal effect plots of power, inequality, and civil liberties on admiration. X-axis is the moderator variable with a histogram of the variables’ distribution. The y-axis is the effect of the predictor variable on admiration at different levels of the moderator variable. The grey band around the slope is the 95% confidence interval. A null effect is highlighted with the dashed horizontal line.*


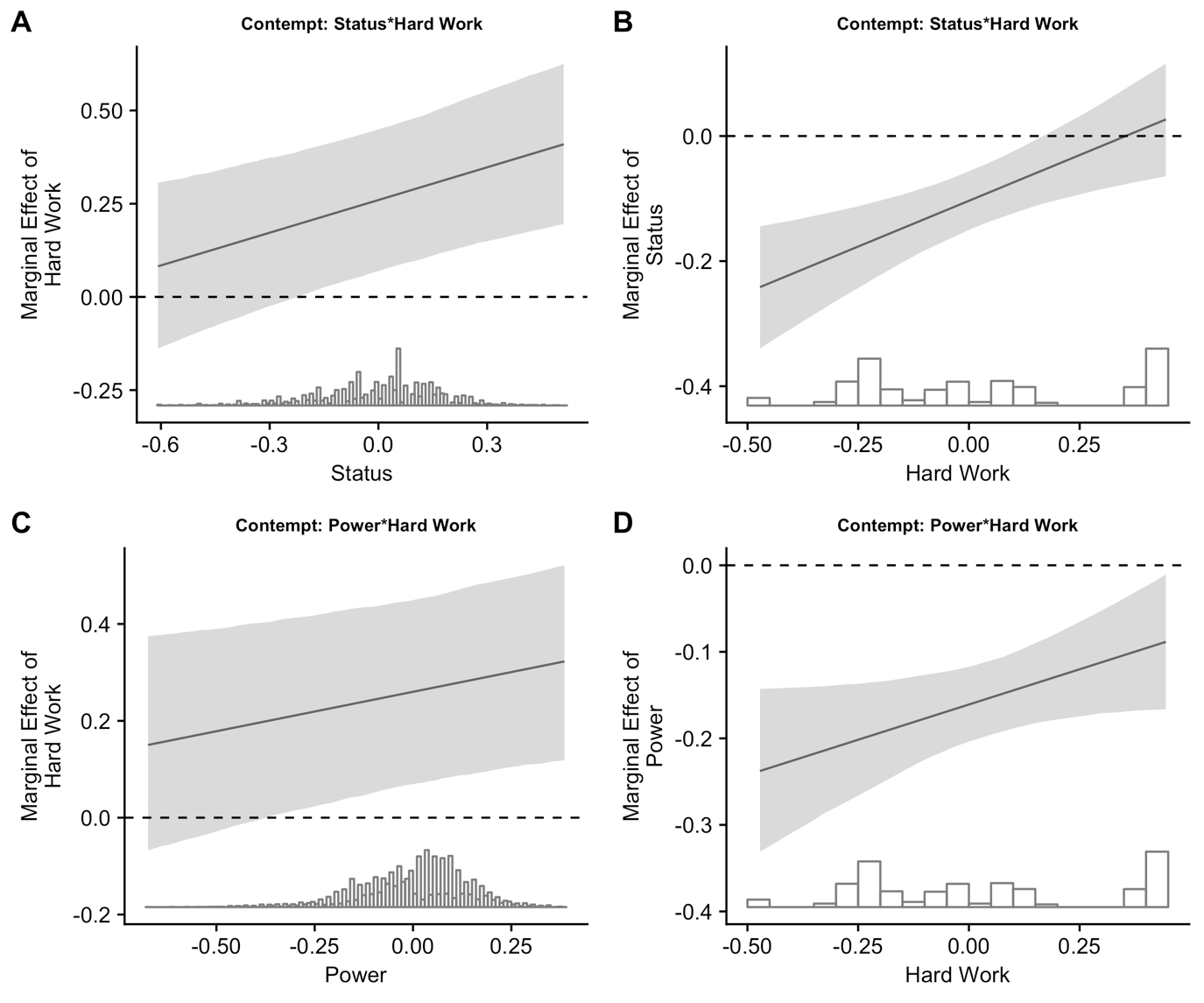


*Figure S13. Marginal effect plots of status, power and hard work on contempt. X-axis is the moderator variable with a histogram of the variables’ distribution. The y-axis is the effect of the predictor variable on contempt at different levels of the moderator variable. The grey band around the slope is the 95% confidence interval. A null effect is highlighted with the dashed horizontal line.*

# Effect of group salience

We manipulated the salience of group interests. The idea was that group salience would have an effect similar to that of identification. We manipulated the salience of group interests by randomly assigned participants in some samples to complete the group-related perceptions (i.e., all of the individual-level moderators and predictors above) *before* the measures of perceived legitimacy or *after* the measures of perceived legitimacy (*N* = 9259, 2698 men, 6521 women, *M*_age_ = 24.3, *SD*_age_ = 9.3). This manipulation was not used in all of the samples because some samples used paper and pencil questionnaires and other collaborators did not have the tools to easily manipulate this variable. These samples are not included in the manipulation analysis. Because perceived legitimacy of the status hierarchy was part of the block included in the manipulation, it was not included as an outcome variable in these analyses.

After data collection had begun, but before the results were known, some of our collaborators argued that the manipulation is not as strong as it could be because, regardless of the experimental condition, participants’ demographic information (and hence status and sense of power) was assessed prior to perceived legitimacy. Although this is the same order used in at least one study that has found support for the power-legitimacy hypothesis (Van der Toorn et al, 2015, Study 2), the order may be enough to make group interests salient, but we cannot assess that possibility here. Therefore, we removed it from the main text and report it here instead. The results are in S14. There is a tiny main effect of group salience, but no significant interaction effects.


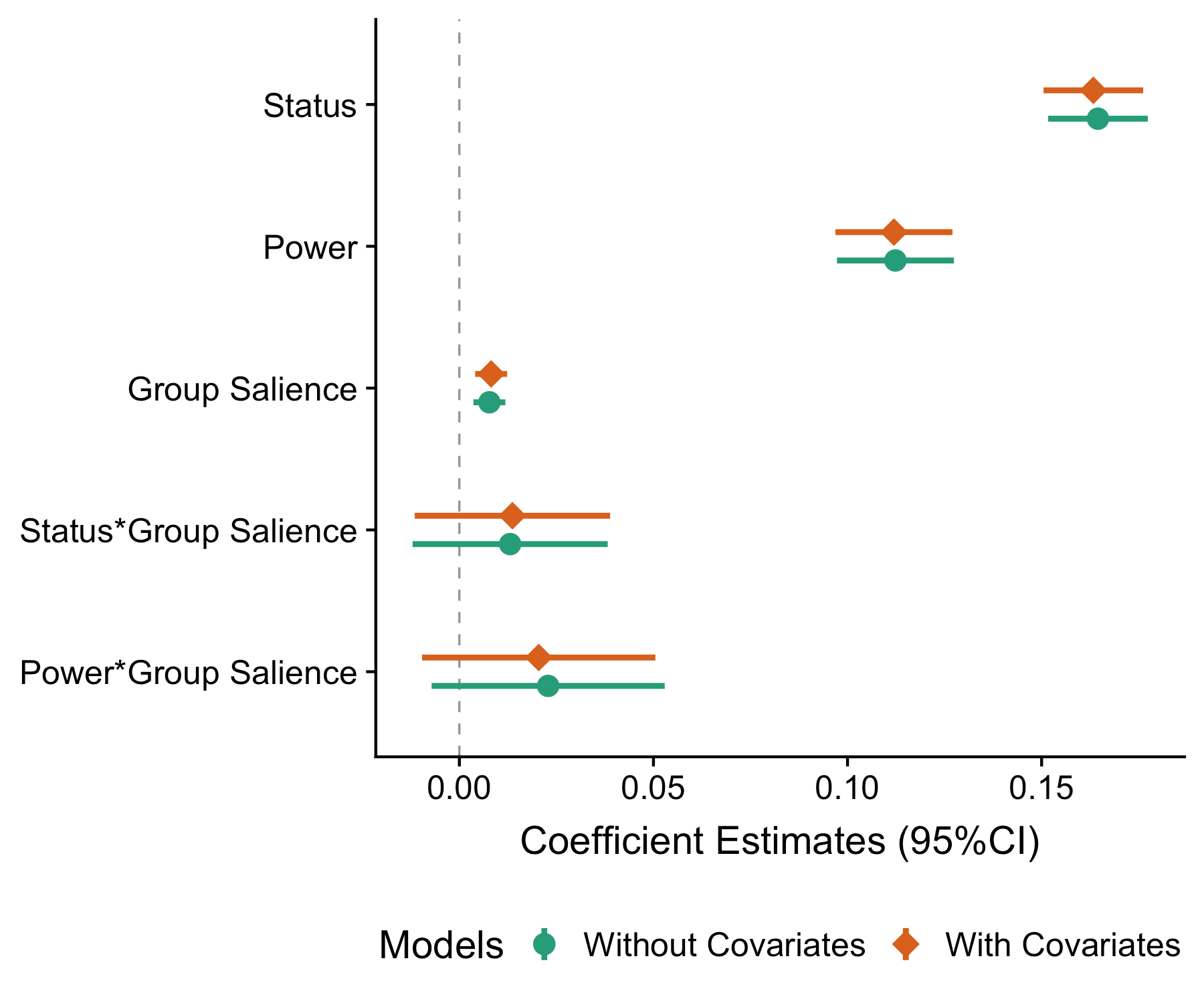


Figure S14. *Effects of status, sense of power, and experimentally manipulated group salience on perceived legitimacy.*
